# Supplementary material for: Cohesin-mediated interactions organize chromosomal domain architecture
Source: EMBO J. 2013 Nov 1;32(24):3119–29. doi: 10.1038/emboj.2013.237 (PMC4489921; doi:10.1038/emboj.2013.237)
Supplement: Supplementary file 1 — Supplementary Information [file embj0032-3119-sd1.pdf]

SUPPLEMENTARY METHODS - to accompany Sofueva/Yaffe *et al.* "A functional role for cohesin in chromosome domain structure"

Cell culture. Polyclonal neural stem cell (NSC) populations were generated as described [1] from $Rad21^{WT/WT}$ and $Rad21^{Lox/Lox}$ mouse ES cells carrying the Cre-ERT2 fusion protein and subsequently cloned to generate individual clonal lines. In the $Rad21^{Lox/Lox}$ strain, *Lox* sites flank exons 5 and 6 of the *Rad21* gene. NSC cultures were maintained as an adherent monolayer in DMEM/Ham's F-12 media with L-glutamine (PAA, Pasching, Austria) containing 0.5x N2 (PAA), 0.5x B27 (Invitrogen, Carlsbad, CA), 50 nM 2-mercaptoethanol (Invitrogen), 120 μg/ml BSA, 4.5 mM HEPES buffer solution (PAA), 1x non-essential amino acids (PAA), 0.15% glucose (Sigma-Aldrich, St. Louis, MO), 1x penicillin/streptomycin (PAA), 10 ng/ml murine EGF (Peprotech, Rocky Hill, NJ), 10 ng/ml human FGF (Peprotech), and 10 ng/ml Laminin (Sigma). NSCs were differentiated into astrocytes (AST) by withdrawing EGF from the media, decreasing the concentration of FGF to 1 ng/ml and adding 10 ng/ml human BMP4 (R&D Systems, Minneapolis, MN). Cells were harvested by treatment with accutase (PAA) for 3-10 min at room temperature followed by a wash with fresh media and centrifugation at 1200 rpm for 5 min at room temperature. Rad21 was depleted by adding 200 nM OHT to NSCs for 48 hrs or 10^7 PFU per ml of growth media Adenovirus-CMV-Cre (Adv-Cre; Vector Biolabs, Philadelphia, PA) to AST cultures for 96 hrs. NSCs were differentiated into ASTs in the presence of BMP4 for 24 hrs before addition of Adenovirus-CMV-Cre to ensure the cells were post-mitotic. Deletion of Rad21 was monitored by genomic DNA qPCR (DNA), RT-PCR (RNA) and Western blotting (protein) levels.

Cell sorting and flow cytometry. To enrich for G_1 NSCs, cells were harvested and fixed with 1% formaldehyde for 10 min as described below for Hi-C. Following two PBS washes, the pellet was resuspended in PI buffer (100 μg/ml propidium iodide, 200 μg/ml RNaseA and 0.05% Triton X-100 in PBS) and left at room temperature for 30 min. Cells were sorted into PBS+1% BSA and pelleted. The pellet was processed further as described for Hi-C. For flow cytometry analysis of cell cycle distribution, cells were fixed with 70% ethanol, pelleted and stained with PI buffer as described above.

Immunofluorescence. Cells were grown in 6-well plates on glass coverslips pre-coated with Laminin. Cells were fixed with 4% paraformaldehyde (PFA) for 10 min, washed twice with PBS, and permeabilized with 0.1% Triton X-100 for 20 min. Cells were blocked with 3% goat serum for 15 min. Primary and secondary antibodies were each incubated for 30 min at room temperature followed by three 15 min washes with

PBS+0.1% Triton X-100. Coverslips were washed with PBS and mounted with Vectashield containing DAPI (Vector Labs, Burlingame, CA). Where indicated, actin was stained with phalloidin for 20 min during the last wash. Primary antibodies: rat anti-mouse Nestin (DSHB Rat 4015; a gift from S.M. Pollard, 1:20), rabbit anti-mouse Olig2 (Millipore, 1:400), rabbit anti-mouse Ki67 (Sigma, 1:500), mouse monoclonal GFAP (Sigma, 1:1000), rabbit anti-mouse Rad21 (Santa Cruz, 1:200), mouse monoclonal Lamin A/C (Santa Cruz, 1:100). Secondary antibodies: anti-mouse Alexa 488, anti-mouse Alexa 568, anti-mouse Alexa 594, anti-rabbit Alexa 488, anti-rabbit Alexa 594, all 1:300 and from Invitrogen.

ChIP-seq. Formaldehyde (1%) was added to the culture medium at room temperature, blocked after 10min with 0.125M glycine, and cells were washed in cold PBS. Nuclear extracts were prepared and pellets were lysed in 150mM NaCl, 25mM Tris-HCl (pH 7.5), 5 mM EDTA, 1% Triton-X, 0.1% SDS, 0.5% deoxycholate for 30 min on ice. Chromatin was sonicated to an average fragment size of 500bp, centrifuged at 13,000 rpm at 4°C to pellet debris, incubated at 4°C with BSA-blocked magnetic PGS beads (Dynal) for 2 hrs, and then overnight with PGS beads coated with rabbit anti-Rad21 antibody (Abcam). The beads were sequentially washed for 5 min with low-salt buffer (0.1% SDS, 1% Triton-X, 2 mM EDTA, 20 mM Tris-HCl, 150 mM NaCl), high-salt buffer (0.1% SDS, 1% Triton-X, 2 mM EDTA, 20 mM Tris-HCl, 500 mM NaCl) and LiCl buffer (0.25 M LiCl, 1% NP-40, 1% deoxycholate, 1 mM EDTA, 10 mM Tris-HCl). Chromatin was RNase A-treated and extracted in elution buffer (1% SDS, 0.1 M $NaHCO_3$). Protein-DNA crosslinks were reversed at 65°C overnight, samples were phenol–chloroform extracted and assessed by qPCR as a quality control. ChIP material was prepared for single-end sequencing on an Illumina GAIIx platform according to protocols provided by Illumina, Inc.

ChIP-seq normalization and definition of genomic landmarks. ChIP-seq reads were mapped to the mm9 genome using bowtie, followed by extension of sequenced tags to 200 bp fragments and pile-up into 50 bp bins. We normalize ChIP-seq coverage by computing the distribution of pile-up coverage on 50 bp bins, and transforming each coverage value v into **–log10(1-quantile(v))**. We denote the normalized values as *ChIP-seq enrichment* or *ChIP-seq intensity* and note that these values are robust to changes in overall sequencing depth, but may be sensitive to different levels of ChIP library purity. To define cohesin and CTCF binding sites, we used a simple threshold on the average values over two biological replicates (T_rad21=2.5 and T_ctcf=2.4). Other selection of thresholds did not change the results significantly. The set of cohesin+CTCF targets analyzed in the paper was derived by intersecting the individual CTCF and Rad21 sites.

To define active and inactive transcription start sites, we analyzed RNA-seq data for control ASTs and NSCs using cufflinks [2] with default parameters along with the GTF file of Ensembl NCBIM37. Active TSSs were set as the top 20% out of all TSSs with mapped reads. This resulted in 8798 and 8863 sites for AST and NSC respectively. Inactive TSSs were defined as UCSC known gene TSSs, which are located 2 K or more away from an active TSS.

CTCF binding energy function. We compute a CTCF DNA-binding energy function from the Cortex CTCF binding sites (ENCODE Cortex CTCF mouse, GSM769019 [3]). Given a set of genomic sites, we compute for each site the maximal energy value within a 200 bp window centered on the point. In Fig. S8 we show a comparison of the distribution of these binding affinities between various genomic sets.

Genome-wide chromatin conformation capture (Hi-C) and library preparation. Hi-C libraries were prepared from freshly accutased cell culture preparations of 10 million cells, which were fixed with 1% formaldehyde for 10-30 min and quenched using 0.125 M glycine. Cells were washed in cold PBS twice, centrifuging the sample at 2500 rpm at 4°C between washes. Pellets were resuspended in 5 ml Hi-C lysis buffer (10 mM Tris HCl pH8.0, 10 mM NaCl, 0.2% NP-40, 1x EDTA-free protein inhibitors (Roche, Switzerland)) and incubated on ice for 20 min. Nuclei were spun down at 1800 rpm at 4°C for 5 min, pellets were resuspended in 500 μl 1.2x NEB2 (New England Biolabs (NEB), Ipswich, MA) with 0.3% SDS and incubated at 37°C for 1 hr in a thermomixer (950 rpm). 2% Triton X-100 was added to the nuclei and incubated as above. Chromatin was digested with 1500 U *Hind*III overnight at 37°C (950 rpm). The following day, nuclei were spun down and incubated in fresh NEB2 buffer containing additional 750 U *Hind*III overnight. The efficiency of digestion was confirmed by visualization on a 1% agarose gel and by using qPCR to amplify across known *Hind*III sites. Only samples with more than 90% efficiency of digestion across at least 5 sites were processed further. To label restriction fragment ends with biotin, each pellet was resuspended in a mixture of 1x NEB2, 15 μM biotin-14-dCTP (Invitrogen), 15 μM dATP, 15 μM dGTP, 15 μM dTTP, and 25 U Klenow polymerase (NEB) in a total volume of 200 μl and incubated at 37°C for 45 min. Proximity ligation was performed *in nucleo,* where nuclei were resuspended in 100 μl 1x T4 DNA Ligase Buffer containing 1600 U T4 DNA ligase (NEB) and incubated at 16°C overnight. Ligation efficiency was verified on a 1% agarose gel. DNA was purified and resuspended in Tris/EDTA buffer. Hi-C libraries were prepared from minimum of 15 μg DNA. To remove biotin-14-dCTP from unligated fragment ends, 5 μg aliquots of Hi-C DNA were mixed with 1x NEB2, 1x BSA, 100 μM dATP, 100 μM dGTP and 5 U T4 DNA polymerase (NEB) in a total volume of 100 μl and incubated for 2 hrs at 12°C. All aliquots were pooled, DNA was purified and

resuspended in TLE Buffer. 4 μg DNA aliquots were sheared using a Covaris sonicator (Covaris, Woburn, MA) under the following settings: duty cycle 20%, intensity 5, cycle burst 200, 140 seconds. Fragment ends were repaired by adding 1x Ligase buffer, 250 μM dNTPs, 15 U T4 DNA polymerase, 50 U T4 Polynucleotide kinase (NEB), and 5 U Klenow polymerase and incubated for 30 min at 22°C. DNA was purified and eluted using the Qiagen MinElute kit (Qiagen; one column per aliquot) and aliquots were run on a 1.5% agarose gel. The gel was stained with 1x SYBR Green (Invitrogen) and visualized under a blue light transilluminator. Material between 200 bp and 400 bp was excised and eluted using the QIAquick Gel Extraction kit (Qiagen). Biotin pull-down was performed using a ratio of 50 μl Dynabeads MyOne Streptavidin C1 beads (Invitrogen) per 5 μg DNA. After washing, the beads were resuspended in 2x No-Tween buffer (10 mM Tris HCl pH 8.0, 1 mM EDTA, 2 M NaCl), pooled with an equal volume of DNA and incubated on a windmill for 1 hr. Non-biotinylated DNA was washed away and blunt-ended DNA fragments were first A-tailed before ligating the Illumina paired-end (PE) sequencing adapters (done according to Illumina protocols). Briefly, beads were resuspended in a 100 μl reaction of 1x T4 DNA Ligase Buffer, 1200 U T4 DNA ligase and 6 pmol adapters per μg of Hi-C DNA (as previously described [4]) and incubated at room temperature overnight. After a series of washes, beads were resuspended in TLE Buffer. Libraries were amplified with a high fidelity polymerase and Illumina PE sequencing primers (PE1.0 and PE2.0) using a cycle number of up to 13. DNA was purified using AMPure XP beads (Beckman Coulter). 1 μl of library material was run on a Bioanalyzer DNA 1000 chip (Agilent) as quality control. Libraries were quantified using the Qubit dsDNA HS quantitation system (Invitrogen).

A random ligation (RL) Hi-C library was prepared as a control, where chromatin was digested *in nucleo* as described above and then de-crosslinked overnight with 1 mg/ml Proteinase K at 65°C before being re-ligated in a 100 μl volume as above.

Hi-C libraries used in this study

Type	Genetic background	Intra-chromosomal	Inter-chromosomal	Total reads
NSC repl. 1	*Rad21*$^{Lox/Lox}$	117,535,500	20,465,212	305,198,160
NSC repl. 2	*Rad21*$^{Lox/Lox}$	109,149,010	19,412,080	276,540,690
NSC *Δ/Δ* repl.1	*Rad21*$^{Δ/Δ}$	86,520,720	18,225,556	214,810,032
NSC *Δ/Δ* repl. 2	*Rad21* $^{Δ/Δ}$	85,581,086	15,150,596	319,616,004
NSC RL	*Rad21*$^{Lox/Lox}$	38,001,556	131,593,206	402,734,922
NSC G_1	*Rad21*$^{Lox/Lox}$	111,449,074	21,603,934	277,856,870
NSC WT	*Rad21* $^{WT/WT}$	28,470,740	5,014,310	64,307,108
NSC WT+OHT	*Rad21* $^{WT/WT}$	24,461,092	3,930,800	56,635,432

NSC (clone 2)	*Rad21*$^{Lox/Lox}$	48,358,656	12,634,970	120,038,548
AST repl. 1	*Rad21*$^{Lox/Lox}$	84,131,500	16,926,498	459,280,174
AST repl. 2	*Rad21*$^{Lox/Lox}$	72,851,536	17,820,360	331,559,222
AST *Δ/Δ* repl. 1	*Rad21*$^{Δ/Δ}$	184,028,156	59,529,742	749,928,938
AST *Δ/Δ* repl. 2	*Rad21*$^{Δ/Δ}$	68,701,236	14,435,680	269,290,634
AST WT	*Rad21*$^{WT/WT}$	48,452,652	9,730,964	126,965,902
AST WT+Adv-Cre	*Rad21*$^{WT/WT}$	41,755,946	8,202,608	114,907,010

Hi-C normalization. Hi-C normalization was performed as described [5]. hicpipe code is available on http://compgenomics.weizmann.ac.il/tanay/?page_id=283). Briefly, fragment ends of *Hind*III cuter sites were filtered for >50% genomic mappability. Paired-end reads were mapped to the genome (build mm9), assigned to fragment ends, and classified according to ligation type. GC-content and fragment length correction matrices were inferred from the data. The inferred correction matrices and a fixed linear genomic mappability correction matrix were used to correct the data. Contact enrichment for a pair of genomic intervals is defined as the ratio between the observed and expected number of contacts between the two intervals using a model that takes into account the above technical biases. Importantly, all analysis in the paper was done by grouping together individual fragment-ends, omitting the use of pre-computed genomic bins to ensure maximal resolution is achieved around e.g., Rad21 sites or TSSs.

Domain identification and clustering. Domains were identified and clustered as described [6]. The only difference in parameters is that scaling factors were inferred using fragment ends that are 200 Kb-800 Kb apart, to account for the lower resolution of the mouse map compared to the *Drosophila* map. Domain borders were called using the 95% percentile of the scaling track. A domain-level map, which takes into account the technical biases and the linear distance corrected by the scaling factors, was partitioned into two clusters, separately for each chromosome. Clusters were assigned as passive/active according to LaminB NPC data downloaded from the UCSC genome browser.

Contact band. Given a *band*, defined by two distances d1<d2, the contact band value for a point x for is:

$$B_{d_1,d_2}(x) = O([x-d_2, x-d_1],[x+d_1, x+d_2])/E([x-d_2, x-d_1],[x+d_1, x+d_2])$$

Where O is the observed number of contacts and E is the expected number of contacts between two given intervals given the technical model. By sliding x along the whole genome in 1 Kb steps we compute a one-dimensional track, called the contact band of

d1,d2. To study insulation structure around a set of genomic landmarks, we assigned for each genomic locus the distance from the closest landmark and compute average B values per distance for a set of bands. In Fig. 2 and Fig. S18, the bands used are 10-15,20-30,30-45,40-60,60-90 and 80-120 (kilobases). For color-coding, we centered the average B trend separately for each band, subtracting the average.

Average interaction profiles for a group of genomic landmarks. We grouped *Hind*III fragment ends into classes by associating each end with a genomic landmark (defined below) located up to 5 Kb from it and then grouping all fragment ends associated with landmark of the same class. We defined classes of cohesin sites, active and inactive TSS sites, enhancer sites and a set of control loci. These classes were further classified to sites within active or passive Hi-C domains. Cohesin sites (passive:28100, active:41811), active TSSs sites (passive:11623, active:24289), CTCF only sites (without Rad21, passive:37490, active:36821) and inactive TSSs sites (passive:25725, active:18161) were defined as described above. Enhancers sites were defined as the union of the top P300 and H3K27Ac ChIP-seq hits (using the top 0.1% quantile, (data provided from B.Martynoga and F.Guillemot) generating 21038 passive and 38109 active loci). The remaining fragment end (not classified given other landmarks) were defined as the background (passive:719089, active:357462).

Given two landmarks L_1,L_2 we define the observed $O_{L_1,L_2}[b_1, b_2]$ and the expected $E_{L_1,L_2}[b_1, b_2]$ contact matrices ($\text{for } i = -10, \ldots, -1, 1, \ldots, 10$) to be the number of observed and expected Hi-C contacts between all pairwise combinations of two sets of 20 fixed 5 K intervals, located before and after each of the landmarks. To examine the specificity of interaction between two classes c_1,c_2 of landmarks that fall within a distance band D=[d_1,d_2] we define the *pairwise class contact enrichment matrix*:

$N_{D,c_1,c_2}[b_1, b_2] = \frac{\sum_{L_1 \in c_1, L_2 \in c_2} O_{L_1,L_2}[b_1,b_2]}{\sum_{L_1 \in c_1, L_2 \in c_2} E_{L_1,L_2}[b_1,b_2]}$, for all L_1,L_2 that are with pairwise distances between d_1 and d_2 basepairs. In Fig. 2b we plot this matrix for c_1=c_2=cohesin and d_1=100 Kb, d_2=200 Kb. We note that by computing this matrix around the central landmark interaction point, we derive inherent control for the specificity of the interaction, since any non-synergistic enrichment in the contact intensity (e.g., general increase in Hi-C coverage for a certain point in the genome) is expected to generate an enrichment between the landmark and all genomic bins around the partner landmark. To perform a comparison across multiple bands (as in Fig. 4c,d) or across multiple combinations of classes (as in Fig. 4e), we replace the matrix by a single enrichment value. We define the *pairwise class contact enrichment* C_{D,c_1,c_2} between two classes using two 10 K windows centered on both landmarks. Formally we use only the four central bins of the whole matrix:

$$C_{D,c_1,c_2} = \frac{\sum_{b_1,b_2,L_1\in c_1,L_2\in c_2} O_{L_1,L_2}[b_1,b_2]}{\sum_{b_1,b_2,L_1\in c_1,L_2\in c_2} E_{L_1,L_2}[b_1,b_2]} \text{ for } b_1 = -1,1 \text{ and } b_2 = -1,1.$$

3D DNA FISH. 3D DNA FISH was performed following protocols described in [7]. Briefly, agar stabs of BAC probes were purchased from BACPAC Resources, transformed into DH5α bacteria and DNA was purified from bacterial cultures using Qiagen Maxi Prep Kits (Qiagen). BAC probes were first confirmed using PCR primers designed to a sequence expected to be within the BAC and then labeled using digoxigenin (DIG) and biotin nick translation kits (Invitrogen) at 16°C for 2-6 hrs. The size of the labeled population (200-400 bp) was verified on an agarose gel and the concentration of each probe was measured with the Qubit dsDNA HS quantitation system (Invitrogen). 50 ng each of two probes were precipitated with 20 μg salmon sperm DNA and 4 μg mouse Cot-1 DNA (Invitrogen) overnight at -20°C and resuspended in 18 μl hybridization buffer (50% deionized formamide and 10% dextran sulfate in 2xSSC). To stain a chromosomal domain, 40 ng each of six probes were pooled and precipitated with 50 μg salmon sperm DNA and 12 μg Cot-1 DNA. Before hybridizing to DNA, probes were denatured for 5 min at 95°C, left on ice for 3 min and pre-hybridized at 37°C for 1 hour. Cells were grown on pre-coated coverslips and fixed with 4% PFA for 10min at room temperature. PFA was removed, cells washed twice with PBS (Ca+/Mg+) and permeabilized with 0.5% Triton X-100 for 10min. Following 3 washes with PBS and a rinse in 2x SSC, the samples were treated with 2x SSC+100 μg/ml RNase A for 1 hr at 37°C. Cells were rinsed with 2x SSC and dehydrated by leaving them in 70%, 90%, and 100% ethanol for 2 min each. Coverslips were air-dried and DNA was denatured in 70% formamide in 2xSSC for 15 min at 80°C followed first by rapid cooling in ice-cold 70% ethanol and then subsequent two-minute washes with 90% and 100% ethanol at room temperature. Coverslips were air-dried and incubated on glass slides with 18 μl of the denatured and pre-hybridized probes overnight at 37°C in a humidified chamber. The following day, coverslips were removed from slides and cells were washed 4x 3 min with 2x SSC at 45°C and 4x 3 min with 0.1x SSC at 60°C. Cells were blocked (5% milk powder in 4x SCC) for 30 min at room temperature followed by incubation with primary antibody for 1 hr at 37°C and secondary antibody for 1 hr at 37°C. To increase the biotin signal, samples were incubated a second time with the anti-biotin primary antibody. 3x 2 min washes with 4x SSC+0.1% Tween 20 were performed at 37°C between each antibody incubation and after the last antibody incubation. Coverslips were washed once with PBS and mounted with 18 μl Vectashield with DAPI (Vector Labs, Burlingame, CA). Primary antibodies used for FISH: anti-DIG FITC from sheep (Roche, 1:20), Avidin Texas Red (Vector Labs, 1:500). Secondary antibodies used for FISH: anti-sheep IgG fluorescein (Vector Labs, 1:100), biotin anti-avidin (Vector Labs,

1:100). Images were acquired using a Leica TCS SP5 confocal microscope. Stacks were collected with a z step size of 0.1 μm. Interprobe distances were measured using the Find Spots and Measure Distances features of the Volocity software (Perkin Elmer). Domain volume was calculated using the Surfaces feature in Imaris (Bitplane Inc., South Windsor, CT). Nuclear volume was calculated using the Find Objects feature in Volocity.

BAC probes used in this study

Probe name	BAC	Genomic location
A	RP23-207B13	chr1: 69742062-69948709
B	RP24-266F7	chr1: 71031672-71318988
C	RP23-331I8	chr1: 71508255-71722223
D	RP23-351B2	chr1: 72032318-72228196
E	RP23-73O21	chr1: 73800317-73945059
F	RP23-82C21	chr1: 70736062-70984124
G	RP23-89F16	chr1: 72328064-72558746
H	RP23-214P23	chr1: 71754239-71942605
I	RP23-144N18	chr1: 70111079-70355740

Number of foci/domains analyzed by FISH

Probe pair	Cells	Number of foci Control	Number of foci Knockout
C-D	NSC	165	115
	AST	656	492
B-D	NSC	470	289
	AST	337	312
A-E	NSC	296	272
	AST	215	241
F-G	NSC	116	90
Domain pool	AST	391	395

Number of AST nuclei analyzed for nuclear volume

Strain	Control	Knockout
Lox/Lox	172	208
WT/WT	214	207

4C-seq. Preparation of 4C samples was performed according to the published protocol [8], with the following changes. Nuclei were processed as for Hi-C and chromatin was digested with 750 U *Dpn*II overnight at 37°C, 950 rpm, and for a second night with another 750 U *Dpn*II. Samples were ligated *in nucleo* in 100 μl volume immediately after digestion with 1600 U ligase in 1x T4 DNA Ligase buffer overnight at 16°C. Following decrosslinking and ethanol precipitation, DNA was resuspended in Tris buffer. 5 μg aliquots were digested with 2 U per μg DNA *Csp*6I (Thermo Scientific) overnight at 37°C. The enzyme was inactivated for 20 min at 65 °C, DNA was extracted and precipitated. To promote self-circularization of *Csp*6I restriction fragments as opposed to formation of concatamers, this ligation step was performed in a large volume. Samples were resuspended in a 6 ml reaction mix containing 1x T4 DNA Ligase buffer and 1600 U T4 DNA Ligase. The reaction was incubated overnight at 16°C. DNA was extracted and precipitated as described above, and resuspended in 10 mM Tris HCl, pH 7.5. 4C libraries were amplified using long primers with 20-22 bp homology to the bait sequence and Illumina paired-end adapter flanks. Primer sequences were chosen to viewpoint sites, which were as close as possible to Rad21 ChIP-seq peaks (primers were taken from the genome-wide 4C primer database constructed by van der Werken *et al* (http://compgenomics.weizmann.ac.il/tanay/?page_id=367/). 4C-seq data analysis and normalization was performed and processed as described [8].

4C primers used in this study

Genomic location of Rad21 ChIP-seq peak	Deregulated gene	Distance between primers and Rad21 ChIP-seq peak (bp)	Reading primer	Non-reading primer
chr10: 91770683-91770922	-	77	CCAATGTAGGAAGCAG**GATC**	ACCCATCTGCTTGATTTACA
chr10: 92477147-92477537	-	1358	CCTAAAGCAGTGCTGA**GATC**	CCCAAACTTAAGCCCTTTAT
chr18: 75145805-75146062	-	Primer at Rad21 peak	ACAAAACTGAGGCTTA**GATC**	CCATTGCAGTTTTAAGAACC
chr18: 75299443-75299709	-	608	CCTTACTCTGTGACAG**GATC**	CCAGACTTTACACTGGCTGT
chr17: 51971972 (no peak control)	-	Nearest Rad21 peak is within 55 Kb of primer	AGGGAAGGAACTCAGG**GATC**	AAATCTATGGGACAGTCTGAAC
chr17: 51917233-51917617	-	Primer at Rad21 peak	TTTCTCATGACTGACT**GATC**	CTGACAGGTTTGGCTTACTC

chr1: 138845589-138845718	-	1638	TTTTCCTTGCTTTGAA**GATC**	CAAAACTGAGCACGTATTGA
chr1: 139122073-139122424	-	1779	TCACATCAACATACTA**GATC**	CTTTGAAAACTCCACAGAGG
chr1: 139941114-139941554	-	633	TGTTCATTTGCACTGC**GATC**	CCCAGAAGTCACACCATATT
chr9: 72614518-72614991	-	Primer at Rad21 peak	TCACAGGACTGGTTTT**GATC**	ATCTCTCCCTTTGCTGACTC
chr9: 72939651-72939856	-	2270	CAATGACATAAAGGCA**GATC**	AAGGGTAATGCTTCACTTCC
chr9: 73010812-73011016	-	74	CGGTCTTACACTGAGG**GATC**	TGAAGGCATTAACCAAACTT
chr9: 73405743-73405919	-	570	GAAAGAGAGGCAATGT**GATC**	TTACGGCAGTCACTATGACA
chr15: 54928695-54928985	Deptor Col14a1	123	GATGGAAACTGGGAAT**GATC**	CCTGGAACTCTCTGTGTTTT
chr15: 54944702-54944980	Deptor Col14a1	843 (Deptor promoter)	GGCTACCCTGGCTAGT**GATC**	ATTTGAATTTGAGGGTATGC
chr15: 55137854-55138246	Deptor Col14a1	1182	TCCGTTTCTCTCTGAG**GATC**	CAGGTTGGGGACCTCTTG
chr3: 103224015-103224152	Olfml3	173	CCGCTCCAAGCACTCA**GATC**	CAATGGGAGCCATATGTTAT
chr3: 103542260-103542539	Olfml3	Primer at Rad21 peak (Olfml3 promoter	GGGGCCATCTGTGTTT**GATC**	ACCTTGTGCTGAGCATTCT
chr4: 137407471-137407730	Ece1	1850	GACTGTGAGACCGTGG**GATC**	TGTGAAGTCTAGGACAACCC
chr4: 62222316-62222714	Rgs3	7903	ATGAGCTATGAGACCA**GATC**	GTTTGGAACTCTCTGGAATG
chr4: 62357078-62357368	Rgs3	Primer at Rad21 peak	GAAACTGGAGGTTCTG**GATC**	AGATTGACCAGGATGAAATG
chr4: 62425132-62425352	Rgs3	857	TCTGCCCTCGAGCTGT**GATC**	TCACTCCTTGATTCTCGACT
chr1: 72912381-72912793	Igfbp5	Primer at Rad21 peak	CTGTCACAGGAATGTT**GATC**	CTGATACCGTAGAGTCAGGC
chr1: 72924353-72924924	Igfbp5	Primer at Rad21 peak	CTTCGAAGTCACAACG**GATC**	TTTGATGCTGAGGAAAGACT
chr18: 75456733 (CTCF-only control)	-	Nearest Rad21 peak is within 70 Kb of primer	GTAATCTACATGAGCA**GATC**	TCCTAGGCAGAAGTAACTGTTT
chr18: 36962212 (CTCF-only control)	-	Nearest Rad21 peak is within 60 Kb of primer	TGTGTTTTTGTGGGGA**GATC**	TTTTCTCCAGAGTTCACAAAC

RNA-seq analysis

RNA-seq reads were assigned to genes using the cufflinks tool. Technical replicates were united, resulting, for each gene, in wild type and cohesin-deficient expression levels, measured in RKPM. To account for different variance in expression of high and low transcribing genes, all genes were stratified according to their wild type expression level intro 100 transcriptional groups, and each gene was assigned a z-score relative to its expression group: $z = \frac{d - mean(d_i)}{sd(d_i)}, d = \log_2(ko/wt)$, where d_i is the set of differences for transcriptional group i. Using a threshold of z=2 we classified 770 genes as up-regulated and 992 genes as down-regulated.

To generate Figure 4b, genes were further classified according to distance from the nearest cohesin/CTCF binding site. Background used for figure 4c includes all genes. To generate Figure 4d, we split all gene pairs, separated by 100-200 Kb into two groups according to the presence of one or more separating cohesin/CTCF sites. For each group of pairs, we computed the Pearson correlation of the z-scores. A confidence interval was computed based on Fisher's Z transform.

RNA-seq libraries used in this study

Hours with Adv-Cre	Type	Genetic background	Uniquely matched	Unmatched	Total reads
72	AST rep. 1	*Rad21*$^{Lox/Lox}$	44,991,352	19,626,546	64,617,898
72	AST rep. 2	*Rad21*$^{Lox/Lox}$	99,372,356	28,661,654	128,034,010
72	AST *Δ/Δ* rep. 1	*Rad21*$^{Δ/Δ}$	161,007,519	10,492,593	171,500,112
72	AST *Δ/Δ* rep. 2	*Rad21*$^{Δ/Δ}$	151,628,664	9,953,814	161,582,478
96	AST rep. 1	*Rad21*$^{Lox/Lox}$	73,299,209	23,353,655	96,652,864
96	AST rep. 2	*Rad21*$^{Lox/Lox}$	142,134,841	15,474,155	157,608,996
96	AST *Δ/Δ* rep. 1	*Rad21*$^{Δ/Δ}$	108,848,912	26,527,864	135,376,776
96	AST *Δ/Δ* rep. 2	*Rad21*$^{Δ/Δ}$	140,294,262	22,034,206	162,328,468

Methods References

1. Conti, L. *et al.* Niche-Independent Symmetrical Self-Renewal of a Mammalian Tissue Stem Cell. *PLoS Biol* **3**, e283 (2005).
2. Trapnell, C. *et al.* Transcript assembly and quantification by RNA-Seq reveals unannotated transcripts and isoform switching during cell differentiation. *Nat Biotechnol* **28**, 511–515 (2010).
3. Shen, Y. *et al.* A map of the cis-regulatory sequences in the mouse genome. *Nature* **488**, 116–120 (2012).
4. Lieberman-Aiden, E. *et al.* Comprehensive Mapping of Long-Range Interactions

Reveals Folding Principles of the Human Genome. *Science* **326**, 289–293 (2009).
5. Yaffe, E. *et al.* Comparative analysis of DNA replication timing reveals conserved large-scale chromosomal architecture. *PLoS Genet.* **6**, e1001011 (2010).
6. Sexton, T. *et al.* Three-dimensional folding and functional organization principles of the Drosophila genome. *Cell* **148**, 458–472 (2012).
7. Eskeland, R. *et al.* Ring1B compacts chromatin structure and represses gene expression independent of histone ubiquitination. *Molecular Cell* **38**, 452–464 (2010).
8. van de Werken, H. J. G. *et al.* Robust 4C-seq data analysis to screen for regulatory DNA interactions. *Nat. Methods* **9**, 969–972 (2012).

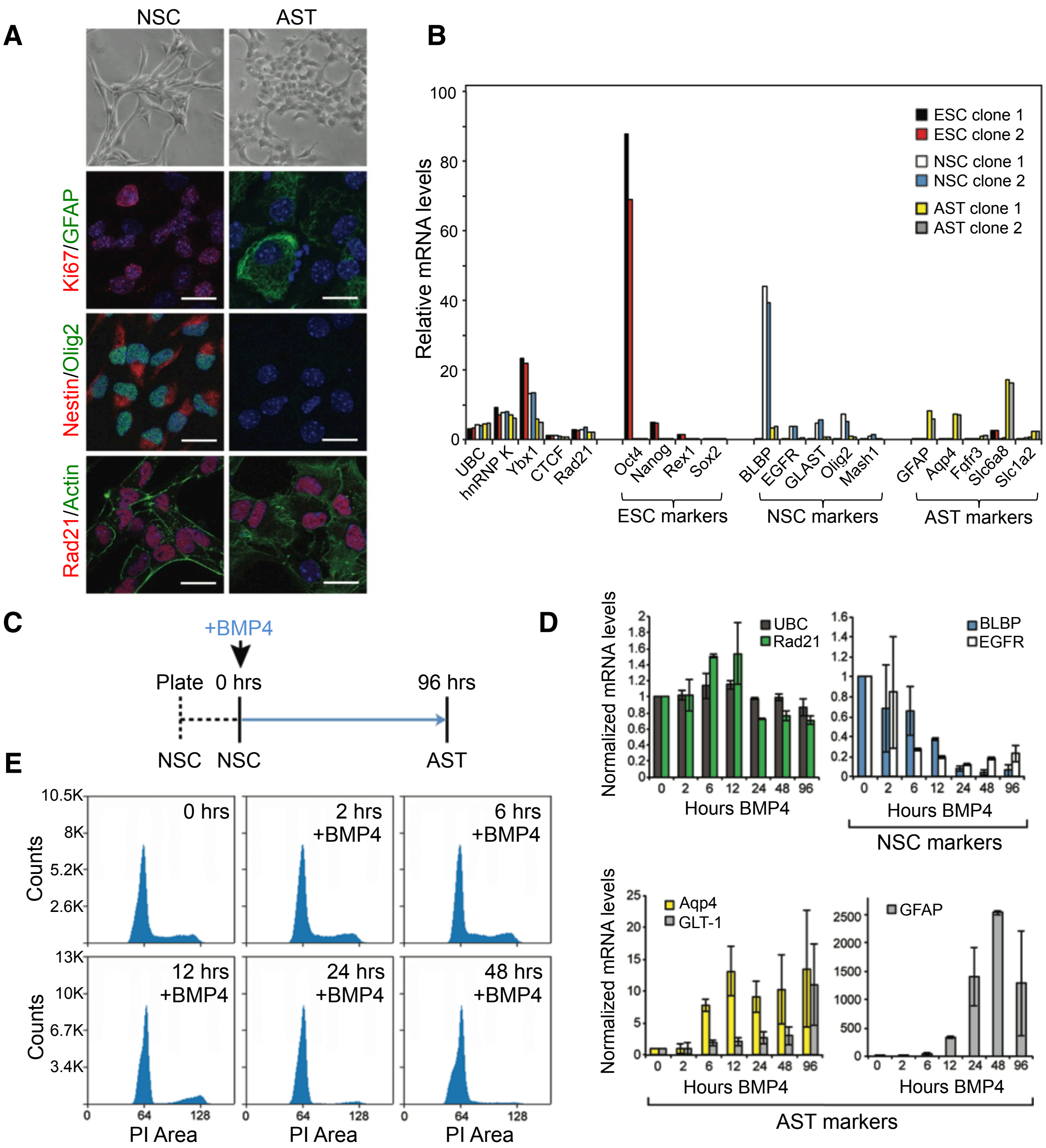

Figure S1: NSC generation and differentiation. A) Representative data showing the differentiation of proliferating *Rad21$^{Lox/Lox}$* NSCs into post-mitotic ASTs. From left to right; phase-contrast images of cellular morphology (magnification 20x); immunocytochemistry for proliferation (Ki67), lineage-specific expression (Nestin/Olig2 in NSCs and GFAP in ASTs), and cohesin (Rad21); counterstain (DAPI). B) qRT-PCR analysis of mRNA levels of lineage-specific genes in ESCs (black/red), NSCs (white/blue) and ASTs (yellow/grey). Two independent polyclonal lines were analyzed. C) Schematic representation of the BMP4 differentiation time course used in this study. NSCs were plated and grown for 24 hrs in the presence of EGF and FGF (dotted line). To induce differentiation, EGF was replaced with BMP4. D) qRT-PCR analysis of the relative expression of lineage-specific genes during a timecourse of differentiation. Data are normalized to the corresponding 0 hr time point (n=3). Note that as NSCs become ASTs, there is a reduction in the expression of the NSC-specific markers (BLBP,EGFR), while expression of AST-specific genes (Aqp4, GLT-1,GFAP) increases. E) Changes in the cell cycle distribution profiles during differentiation. 24 hrs after BMP4 addition, >90% of cells have exited the cell cycle.

Figure S1

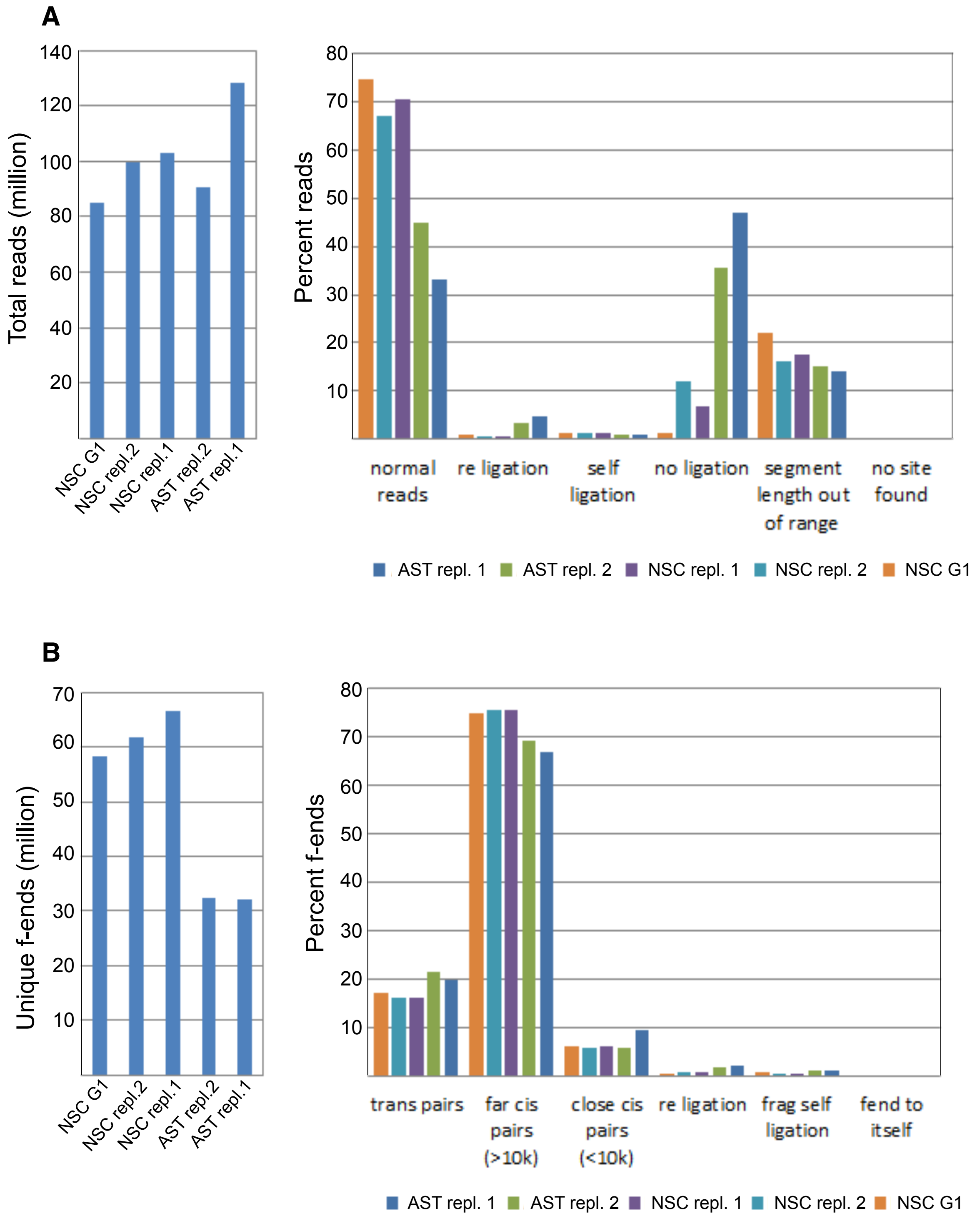

Figure S2: Hi-C pipeline. Hi-C reads were processed as described in Yaffe and Tanay (2011). A) Shown are key read statistics for five Hi-C libraries. 'Normal reads' are valid Hi-C products. Also shown are other products that have been filtered out from further analysis. B) Shown are key statistics on fragment end pairs (f-ends) that have been extracted from the normal reads and used to construct the Hi-C maps. The overall quality of all five libraries is high, as indicated by the good ratio between intra- (cis) and inter-(trans)chromosomal contacts.

Figure S2

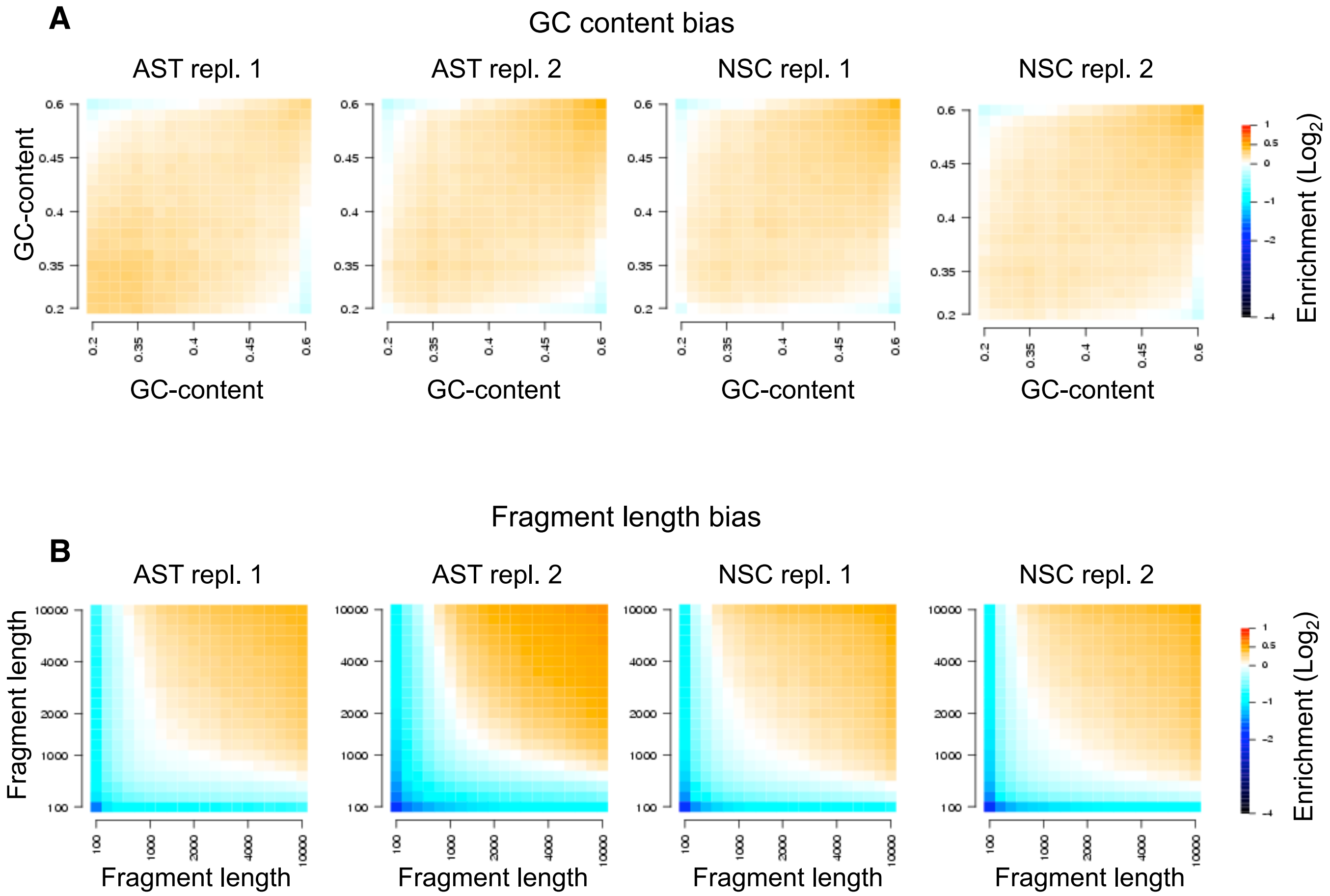

Figure S3: Hi-C bias correction. GC-content (A) and fragment length (B) technical correction matrices generated using the algorithms described in Yaffe and Tanay (2011). These parameters are used to predict the probability of observing contacts in the Hi-C map and are calculated based on sequence alone. Using this probabilistic approach, each pair of fragment ends is assigned with a specific correction factor, which eliminates the need for pre-assumed binning and provides the opportunity for statistical analysis at an arbitrary resolution, as shown in the main text.

Figure S3

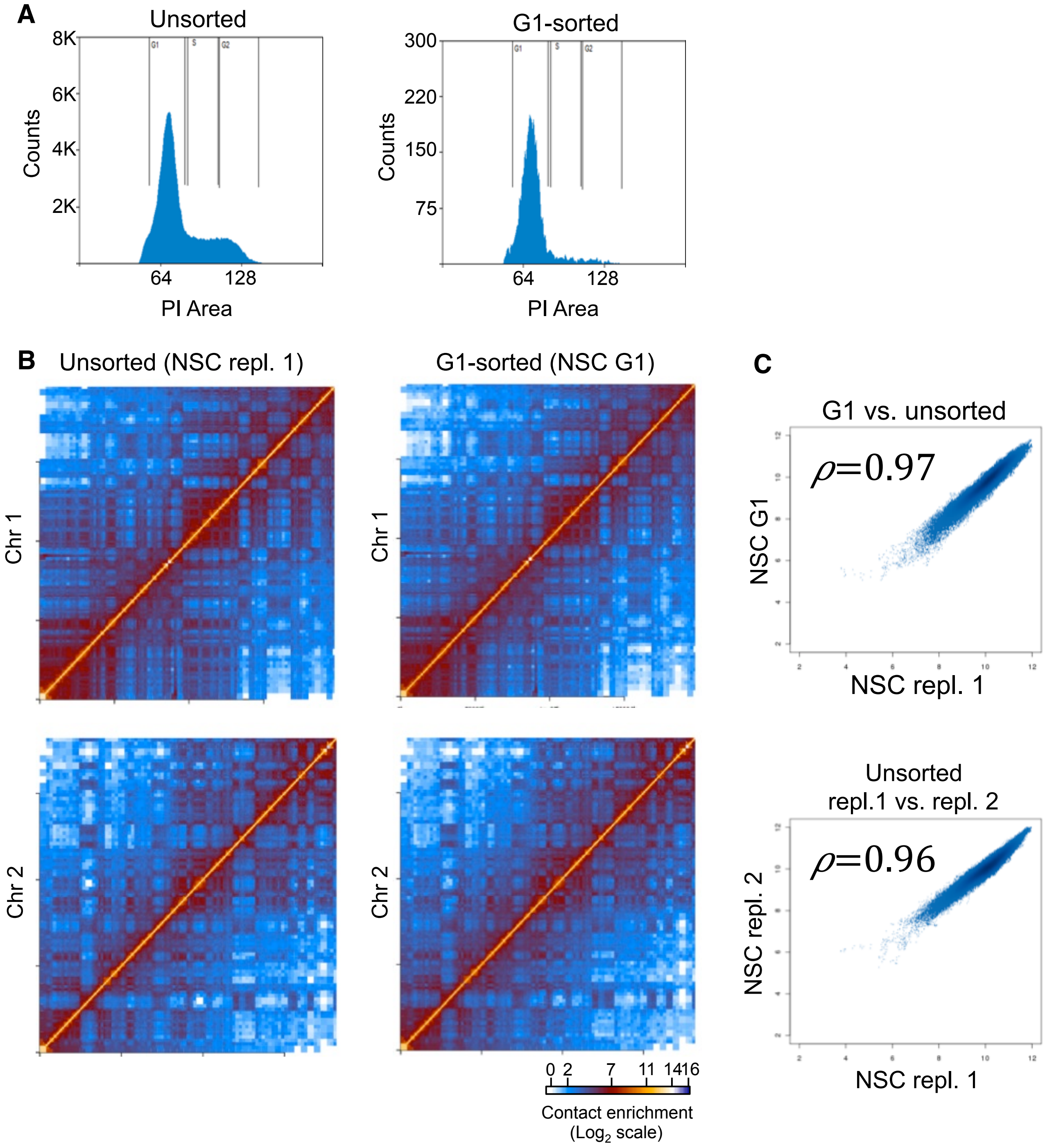

Figure S4: G1-sorted NSCs show similar contact profiles to unsorted NSCs. G1-sorted NSCs were profiled using Hi-C and compared to unsorted NSCs to control for biases arising from cell cycle distribution differences between NSC populations (note the higher percentage of cells in G1 in Rad21-deficient NSC cultures). A) Flow cytometry profiles of unsorted and G1-sorted NSCs. B) Hi-C contact maps for chromosomes 1 and 2. C) Scatter plots depicting the correlation between the unsorted and G1-sorted Hi-C libraries (bottom: correlation between two biological repeat libraries from unsorted NSCs) for contact intensity in the 160-480 Kb band, smoothed in 10 Kb windows. The high correlation between the two Hi-C libraries suggests that such biases do not confound the analysis by profoundly changing the map's contact structure.

Figure S4

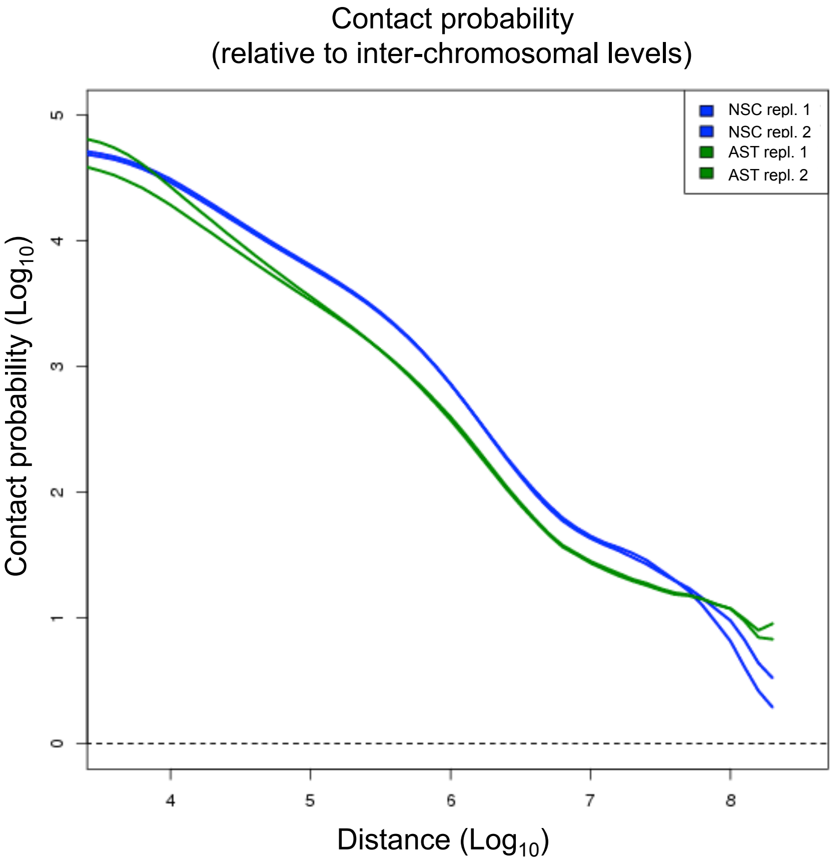

Figure S5: Contact probability as a function of linear distance. Plots showing contact probability (normalized to the inter-chromosomal contact probability) between two fragment ends as a function of the linear intra-chromosomal genomic distance. The trends for NSC and AST are generally similar, with a constant factor distinguishing the maps between 100 Kb and 10 Mb.

Figure S5

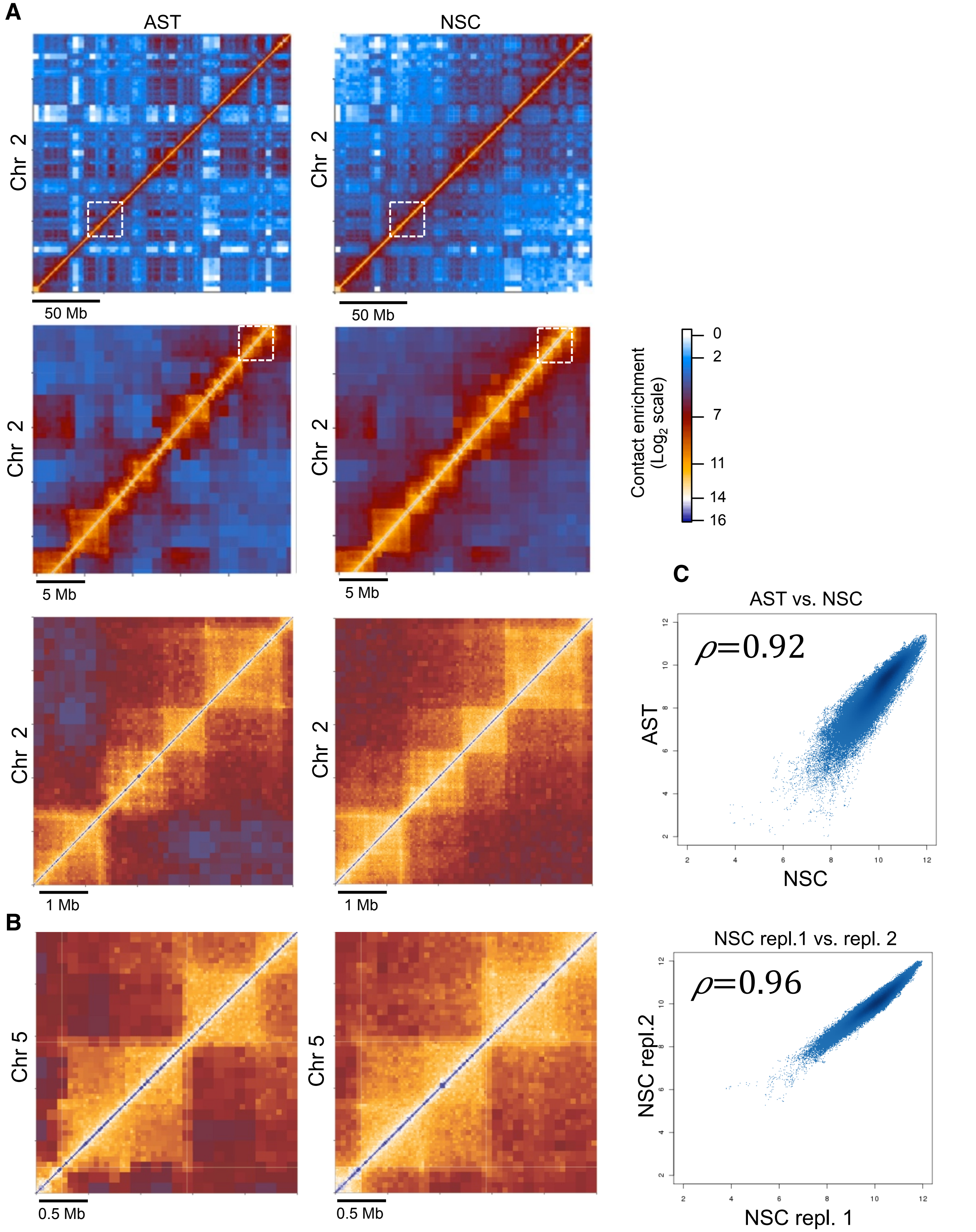

Figure S6: NSC and AST Hi-C maps are structurally similar. A) NSC and AST Hi-C maps of the entire chromosome 2, a 25 Mb region and a 5 Mb region within it. B) NSC and AST Hi-C maps of a 2.5 Mb region on chromosome 5. C) Scatter plots of the 160-480 Kb contact intensity band (methods), smoothed over 10 Kb windows, between AST and NSC libraries and between the NSC replicates.

Figure S6

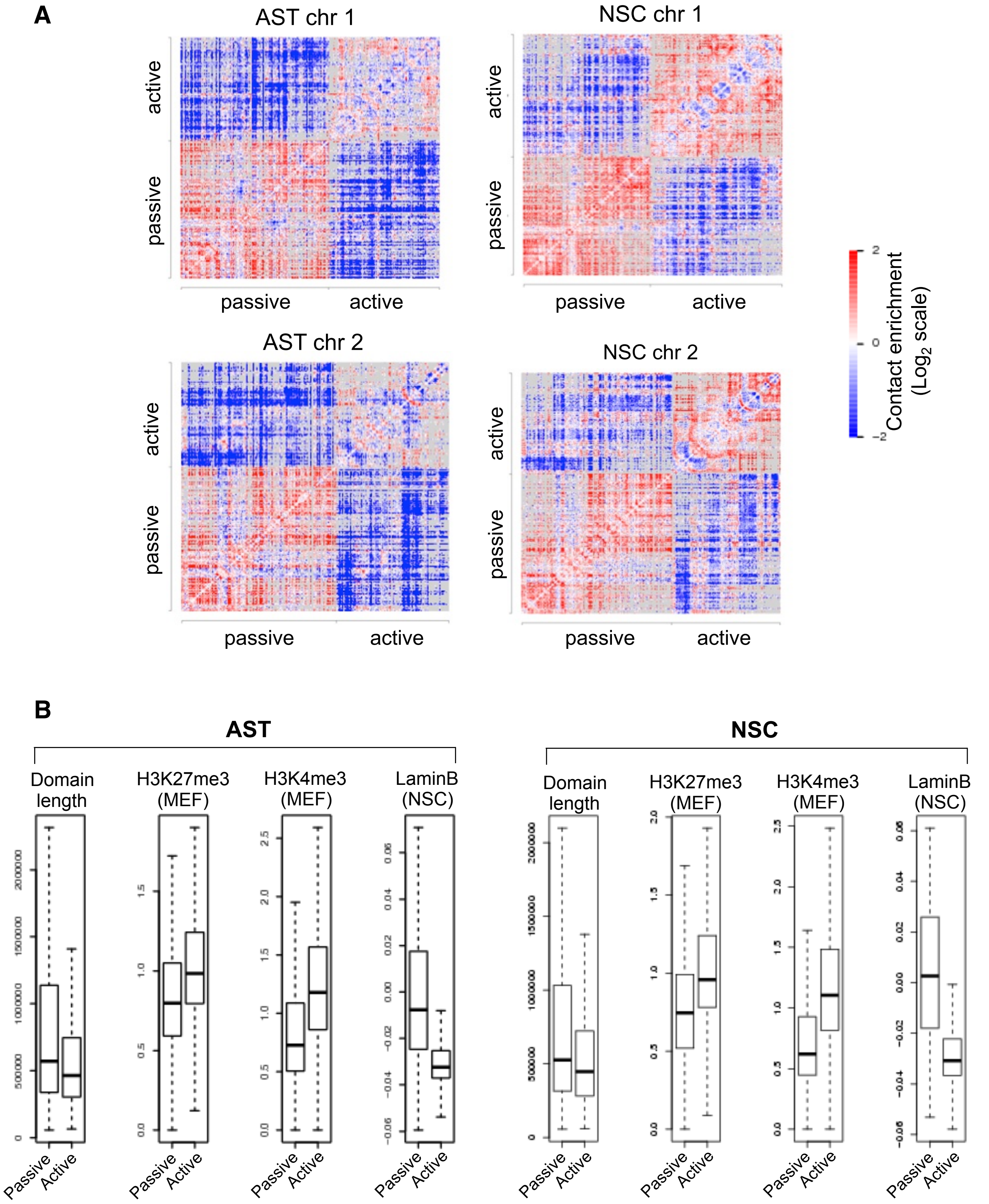

Figure S7: Active-passive domain clustering. Chromosomal domains from the Hi-C maps were divided into two clusters as described in Yaffe and Tanay (2011). A) Clustering results for two representative chromosomes. B) Distributions of domain length and various epigenetic properties of the two domain clusters.

Figure S7

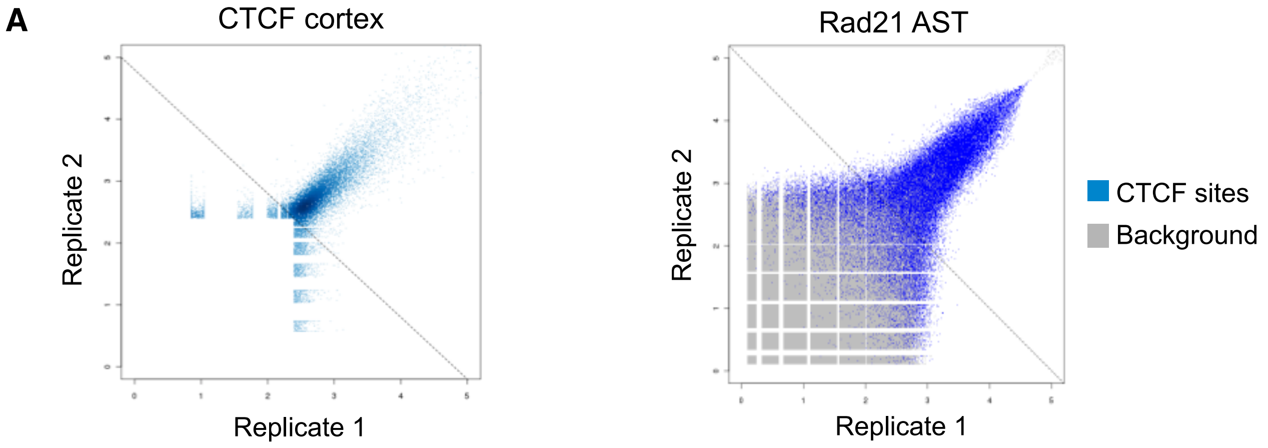

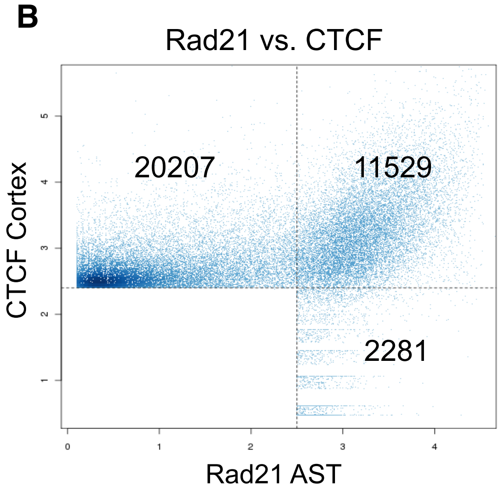

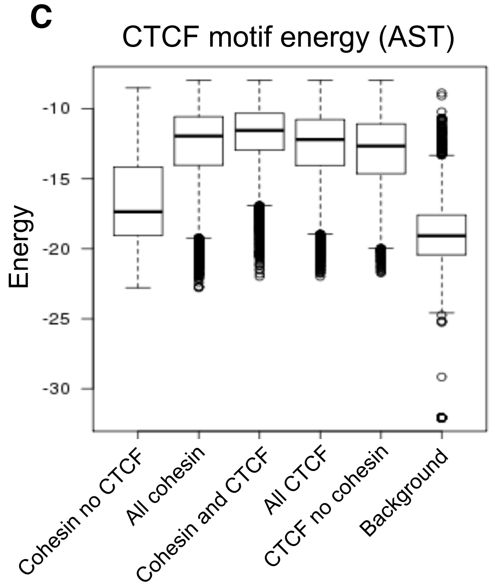

Figure S8: ChIP-seq analysis of CTCF and Rad21 binding sites. A) A comparison of percentile-normalized ChIP-seq replicate libraries of CTCF (left) and Rad21 (right). The cutoff threshold for defining *ad-hoc* CTCF binding sites is shown as a dashed line. The Rad21 ChIP-seq signal in ASTs is colour-coded depending on whether or not it coincides with CTCF ChIP-seq signal (gray to blue). The threshold used to define bone-fide Rad21 binding sites is also marked by a dashed line. B) A comparison of Rad21 and CTCF percentile-normalized ChIP-seq values. The number of peaks associated with each combination of factors is shown. Note that combined Rad21 and CTCF sites are generally of higher intensity than CTCF- or Rad21-specific sites. Note also that the vast majority of Rad21 sites show significant CTCF present but that a large population of CTCF sites lack Rad21 signal in our cells. C) We used a CTCF motif model to compute the sequence affinity of different classes of binding sites to the CTCF binding motif. The boxplot depicts the distribution of CTCF sequence affinity (or motif energy) and indicates that Rad21-free CTCF sites are valid targets for CTCF. D) A comparison of Rad21 ChIP-seq intensities in NSCs and ASTs. We observe a highly similar cohesin binding landscape, consistent with the generally conserved domain and subdomain insulation structure.

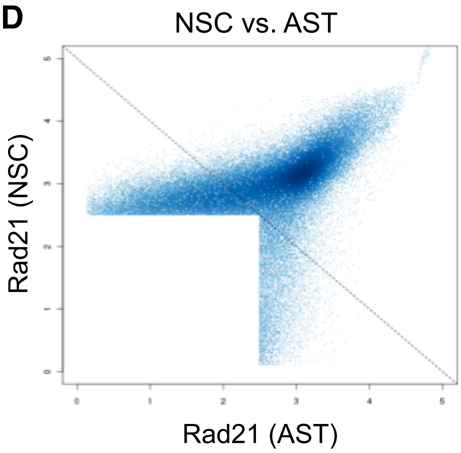

Figure S8

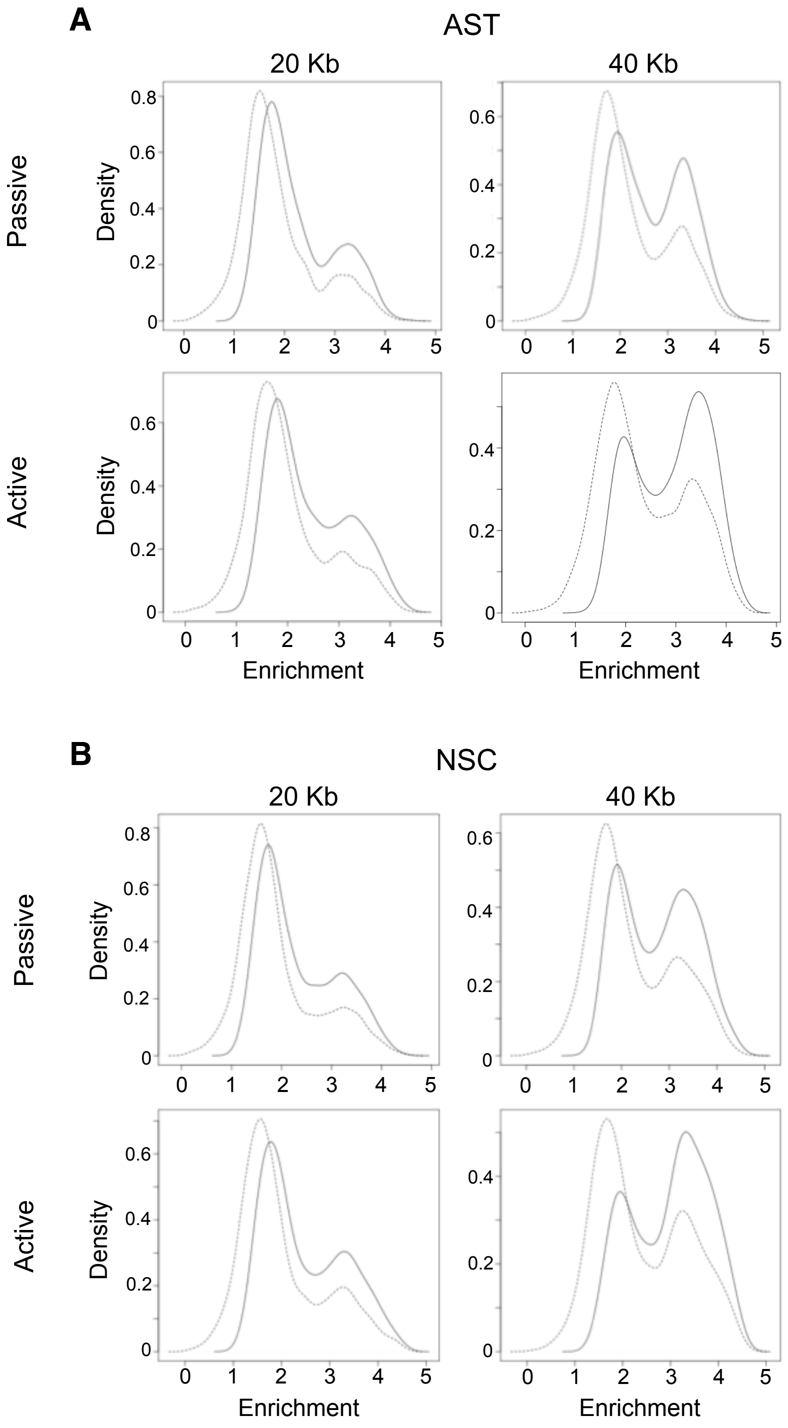

Figure S9: The vicinity of domain borders is enriched for high Rad21 signal. Distribution density of the maximal Rad21 ChIP-Seq value within a given genomic window (20 Kb or 40 Kb) centered on borders between passive (top) or active (bottom) domains in ASTs (A) or NSCs (B). A control with random borders is shown in a dashed line ("Enrichment" on the x-axis pertains to ChIP-Seq enrichment values). We believe that the limited resolution of the Hi-C map in determining the domain border (correct only within 4-5 *Hind*III fragments) prevents stronger enrichment values, such as those observed in *Drosophila*.

Figure S9

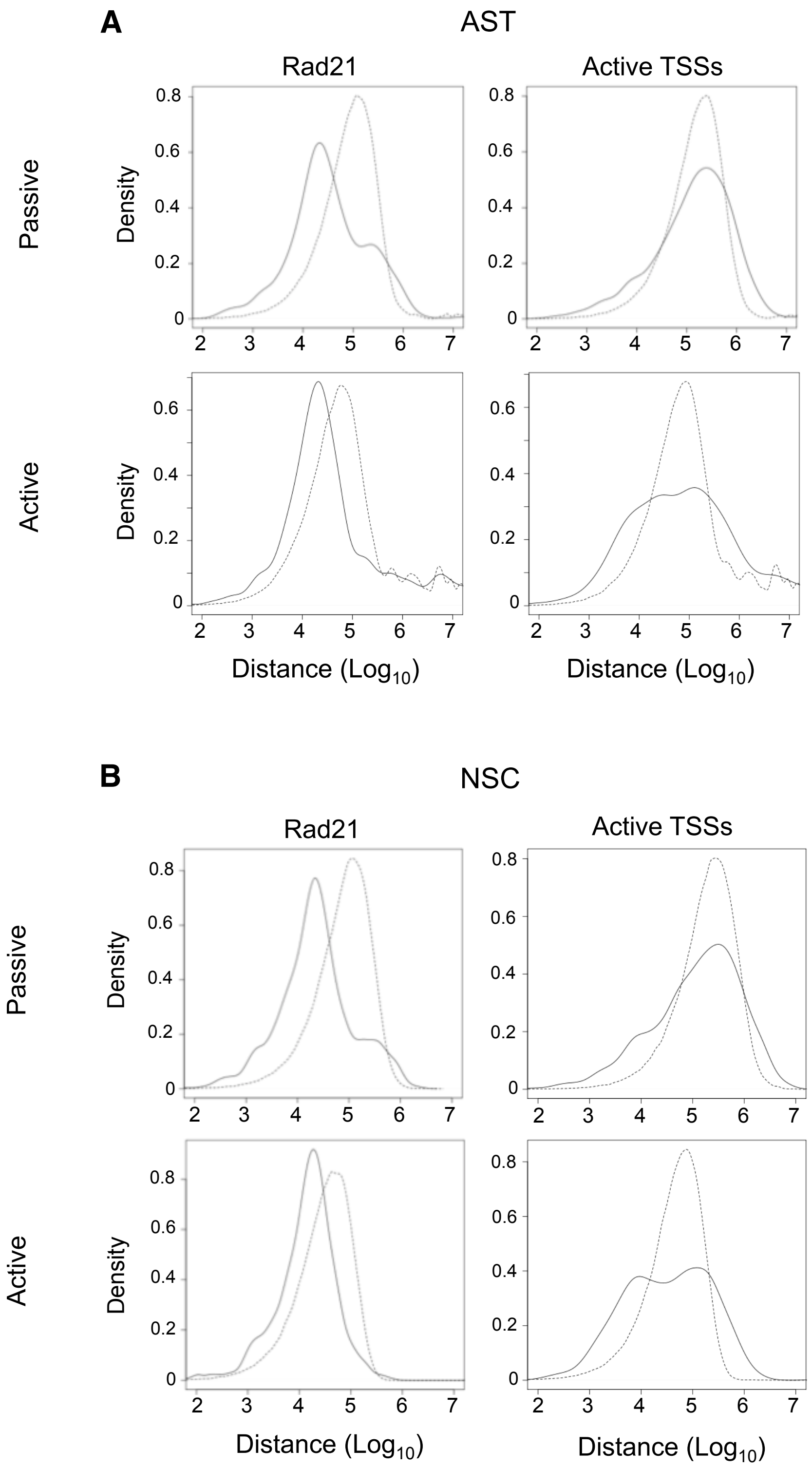

Figure S10: Domain borders are enriched for a nearby cohesin site. Distribution density of the distances between domain borders and their nearest cohesin site or active TSS site in ASTs (A) or NSCs (B). Data is shown for borders separating passive (top) or active (bottom) domains. A random sample of chromosomal regions with a matching size has been used as a control (dashed line).

Figure S10

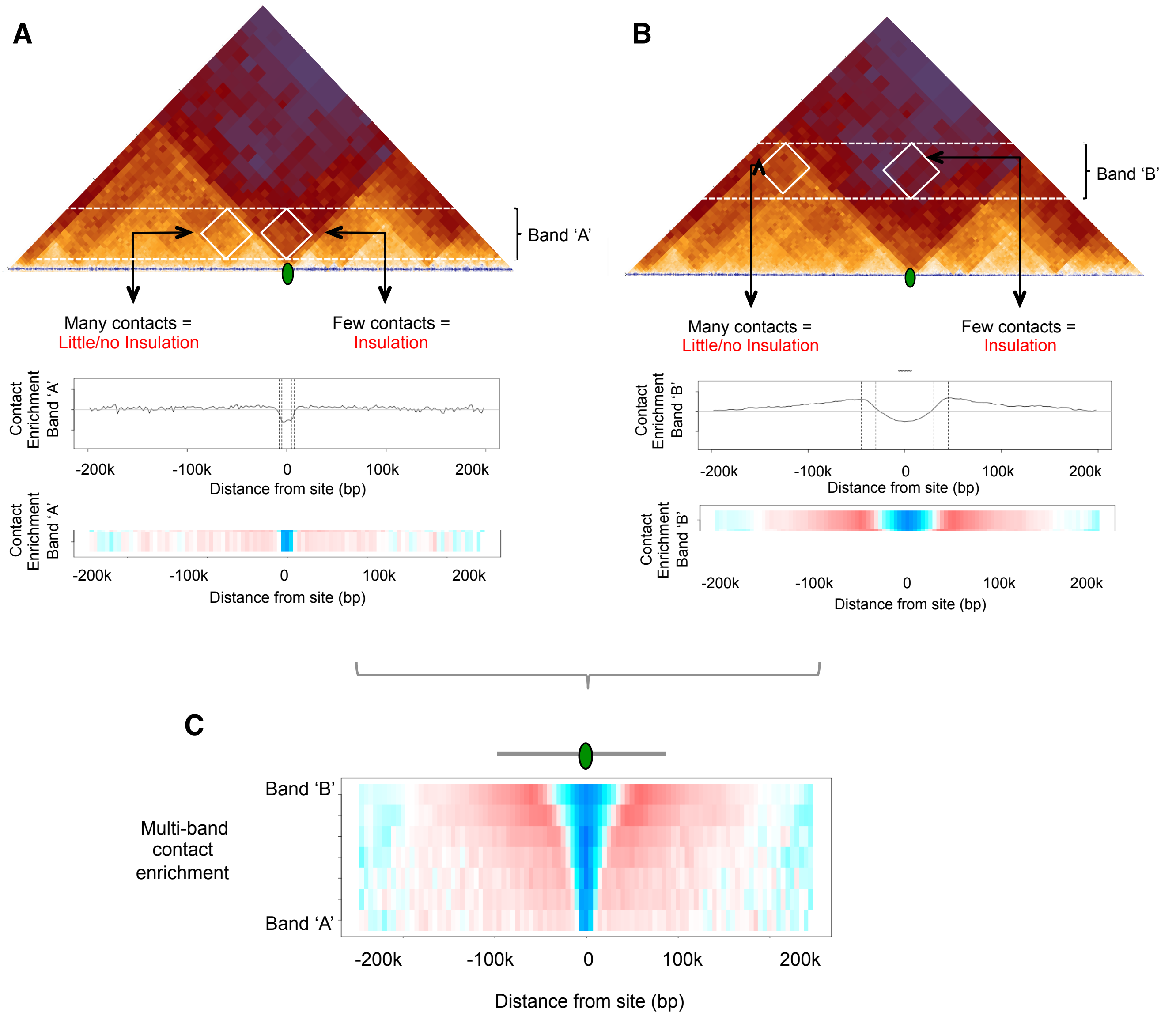

Figure S11: A schematic representation of the method used to calculate contact insulation for a given location in the genome across multiple scales. If insulation is occurring from a given point in the genome (green circle), then you detect fewer contacts crossing the point compared to the neighboring regions. This depletion of contacts represents *contact insulation*. Conversely, if many contacts are detected across a point this represents little or no insulation (compare triangles in Hi-C matrix). Using the Hi-C matrix, we calculate the average intensity of contacts between all chromosomal elements that lie within a certain distance band (Band 'A' or Band 'B' shown here) on either side of a given feature in genome (green circle). This distribution of contact frequency is plotted below the Hi-C matrix and can also be represented as a heatmap to show the normalized contact frequency for a given Band. By repeating this analysis with increasing distances from the given point in the genome, contact insulation can be computed across multiple scales (compare A) and B)). C) These graphs can be collapsed into one heatmap to show the normalized level of contact insulation at multiple scales simultaneously, where red and blue represent an enrichment or depletion of contacts, respectively.

Figure S11

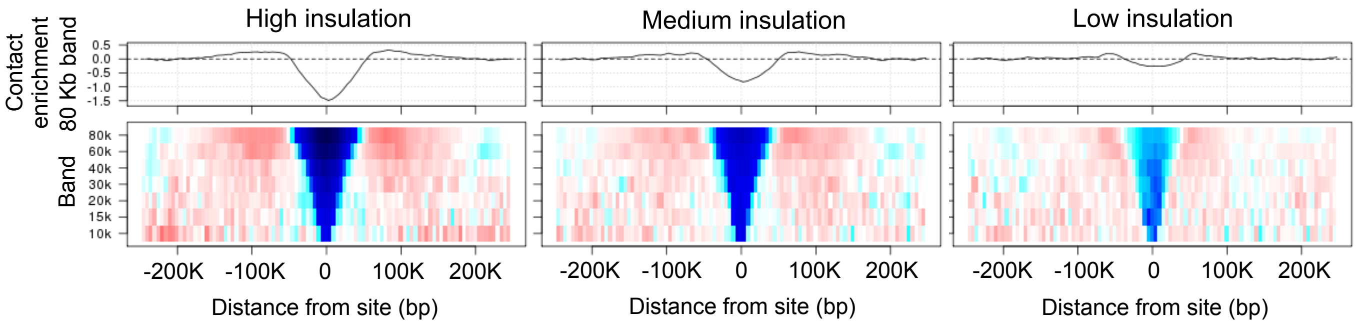

Figure S12: Cohesin/CTCF sites engage in high, medium and low insulation. Cohesin/CTCF sites were grouped according to their extent of insulation (based on their peak-to-trough ratios at the 80 Kb band) and the insulation analysis was then repeated for the top, middle two, and bottom quartiles. Significant insulation signatures were observed for all groups with a particularly strong effect at the 10 Kb band, suggesting that even if cohesin sites do not engage in insulation at larger scales, they are still able to influence their local environment.

Figure S12

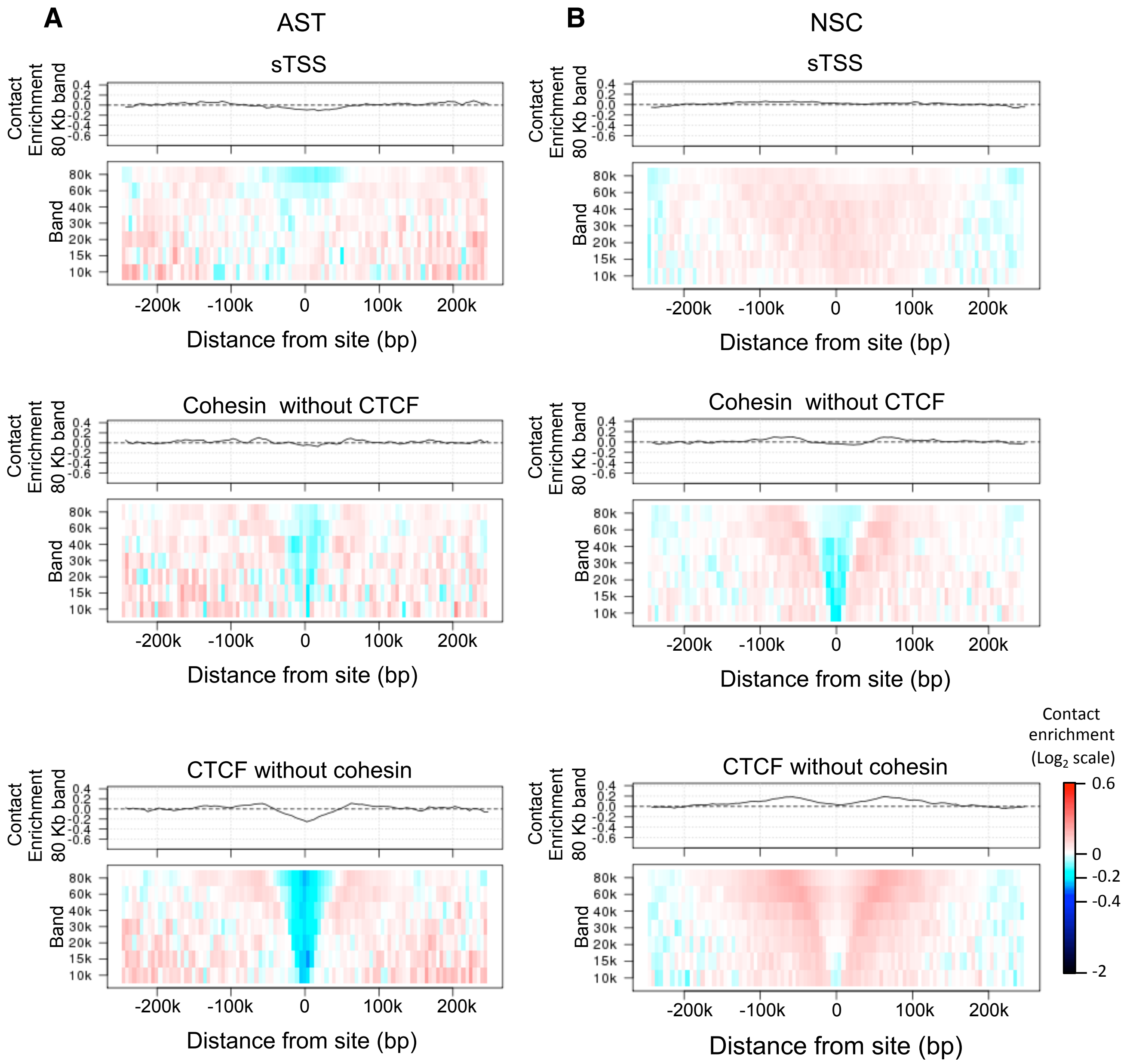

Figure S13: Strong insulation is unique to cohesin sites colocalized with CTCF. Shown are insulation diagrams averaged around silent TSSs, cohesin sites without CTCF and CTCF sites without cohesin (as in Figure 1e) for ASTs (A) or NSCs (B).

Figure S13

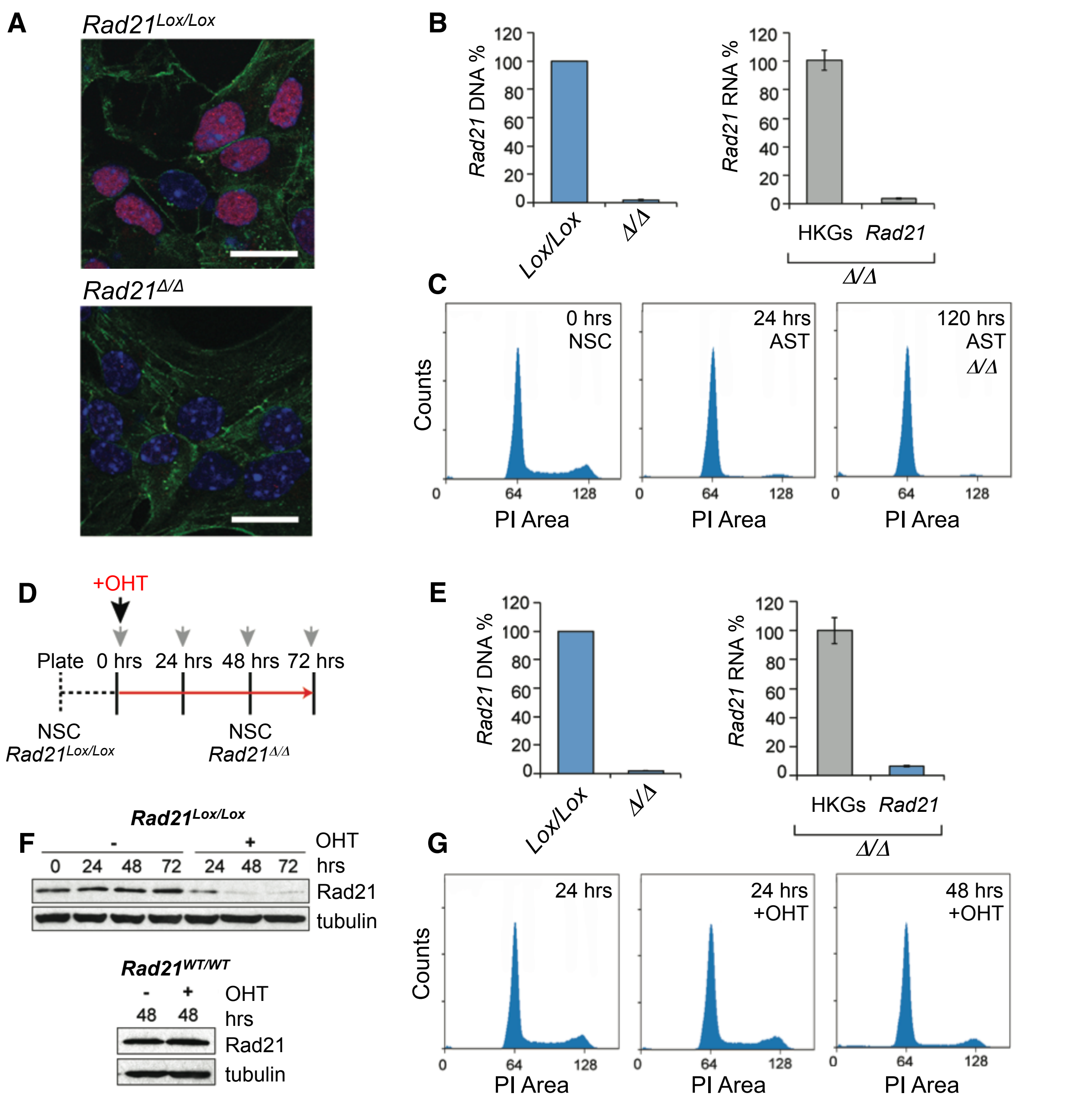

Figure S14: Rad21 depletion in NSCs and ASTs. A) Immunocytochemistry of Rad21 protein in *Rad21*$^{Lox/Lox}$ *and Rad21*$^{\Delta/\Delta}$ ASTs. Scale bar = 20 µm. B) Analysis of *Rad21* genomic DNA and mRNA levels (n=4) in control (*Rad21*$^{Lox/Lox}$*)* and mutant (*Rad21*$^{\Delta/\Delta}$) ASTs to monitor deletion. HKG = housekeeping genes. C) Cell cycle distribution assessed by PI staining and flow cytometry. Loss of Rad21 in ASTs does not adversely affect their cell cycle distribution or viability and the cells remain quiescent. Percentages in each cell cycle phase is as follows: NSCs – dead (3%), G1 (68%), S (15%), G2 (14%); ASTs – dead (4%), G1/G0 (92%), S (1%), G2 (3%); ASTs+Adv-Cre – dead (4%), G1/G0 (93%), S (1%), G2 (2%). D) A schematic representation of the protocol used to deplete Rad21 in *Rad21*$^{Lox/Lox}$ NSCs. NSCs were grown for 24 hrs when OHT was added. Grey arrowheads represent timepoints when samples were collected. E) Analysis as in B) where (n=5) for DNA and (n=3) for mRNA. F) Immunoblot analysis of Rad21 protein levels following OHT addition to *Rad21*$^{Lox/Lox}$ and *Rad21*$^{WT/WT}$ NSCs. Quantification using ImageJ shows that >95% of Rad21 protein is lost after treatment with OHT for 48 hrs. G) Effects of Rad21 depletion on the cell cycle distribution of NSCs done as in C). Percentages in each cell cycle phase is as follows: NSCs (24 hrs) – dead (3%), G1 (68%), S (15%), G2 (14%); NSCs+OHT (24 hrs) – dead (3%), G1 (69%), S (15%), G2 (13%); NSCs+OHT (48 hrs) – dead (7%), G1 (74%), S (8%), G2 (11%).

Figure S14

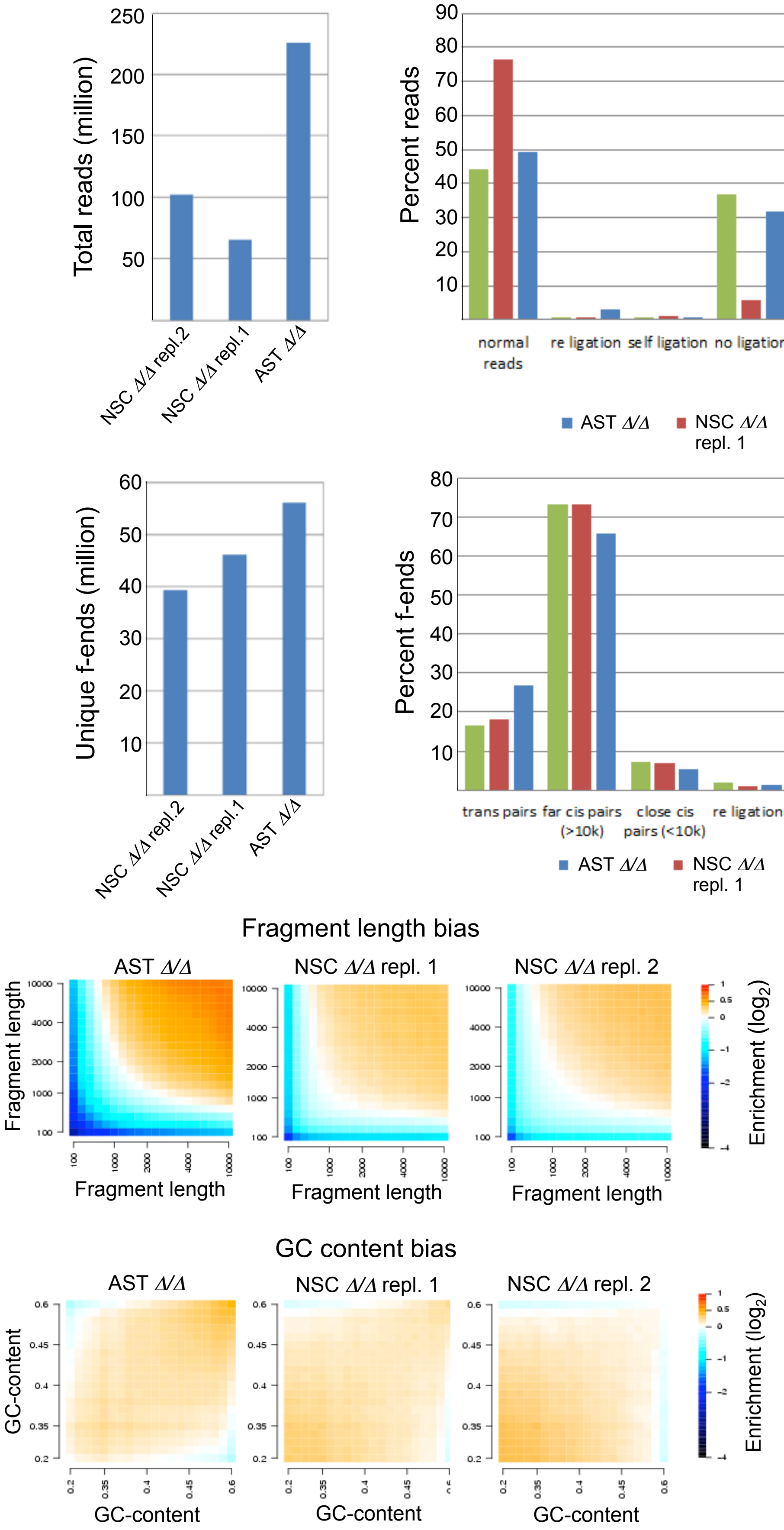

Figure S15: Hi-C libraries from Rad21-deficient cells. Statistics and correction biases are shown for Hi-C maps generated from Rad21-deficient cells. See Fig. S2.

Figure S15

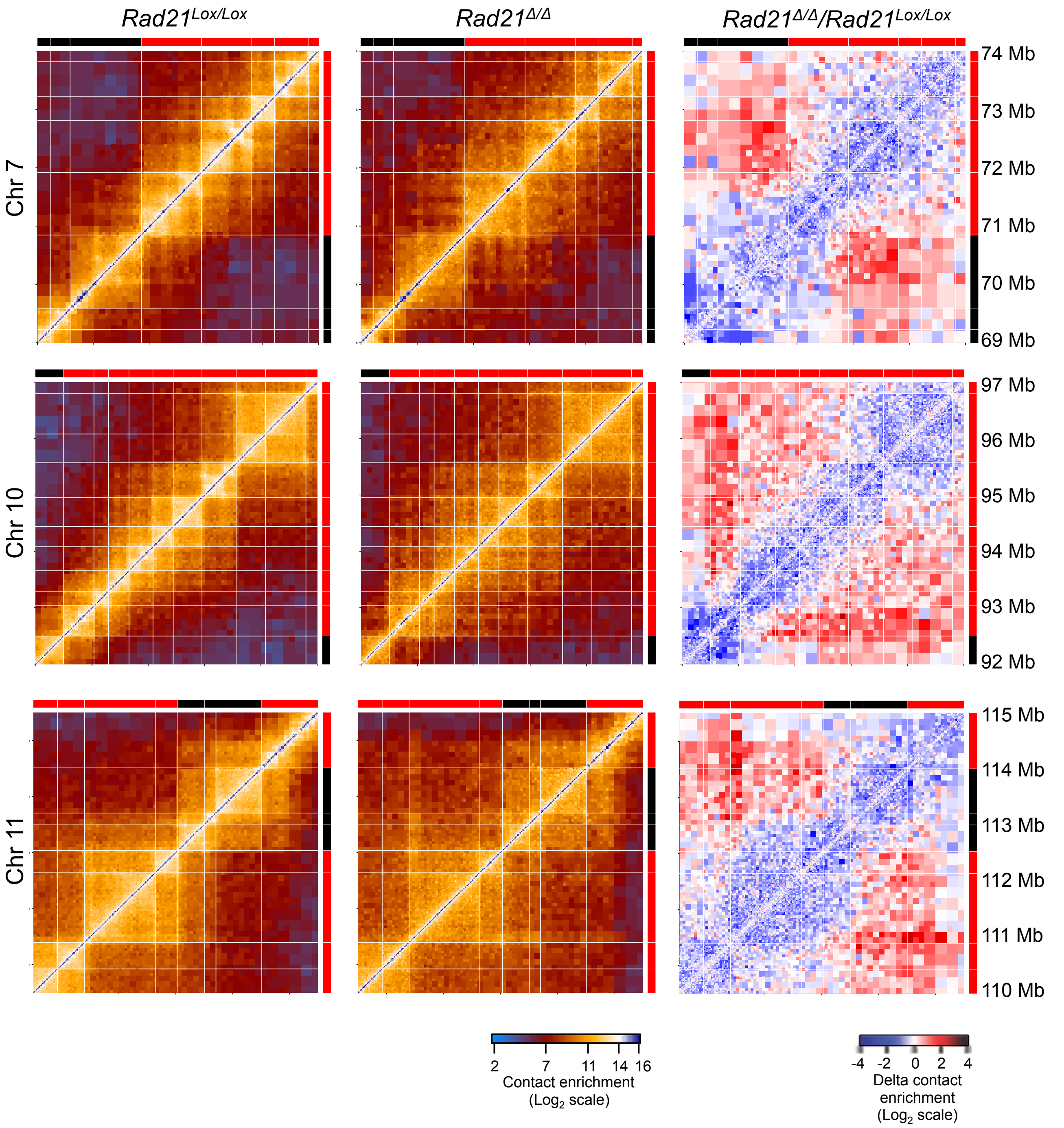

Figure S16: Examples of intra-domain decompaction and novel inter-domain interactions in Rad21-deficient cells. Technically corrected Hi-C contact maps of 5 Mb regions from chromosomes 7, 10 and 11 before and after Rad21 depletion. Also shown is a delta map for each chromosomal region, colour-coded according to the difference in contact intensity between the two conditions. Red and blue represent increased or decreased contacts in *Rad21*$^{\Delta/\Delta}$ compared to *Rad21*$^{Lox/Lox}$, respectively.

Figure S16

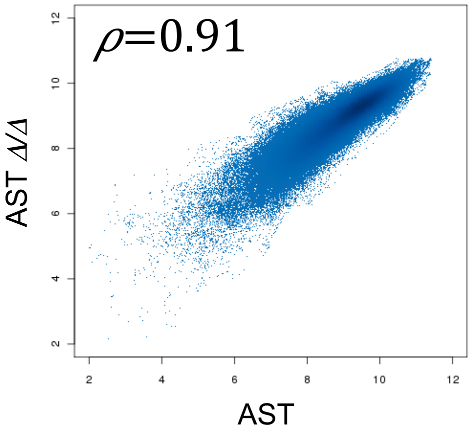

Figure S17: Rad21-deficient cells display structural changes. A comparison of the 160-480 Kb band contact intensities (10 Kb smoothed) calculated from Hi-C libraries prepared from control ASTs and Rad21-deficient ASTs (*Δ/Δ*). High values represent loci with strong cross-connectivity, low values represent possible insulation hotspots. The correlation is significantly smaller compared to replicated experiments but we do not observe major new insulation hotspots (bottom right part of the plot) or loss of major existing insulation hotspots (top left part of the plot).

Figure S17

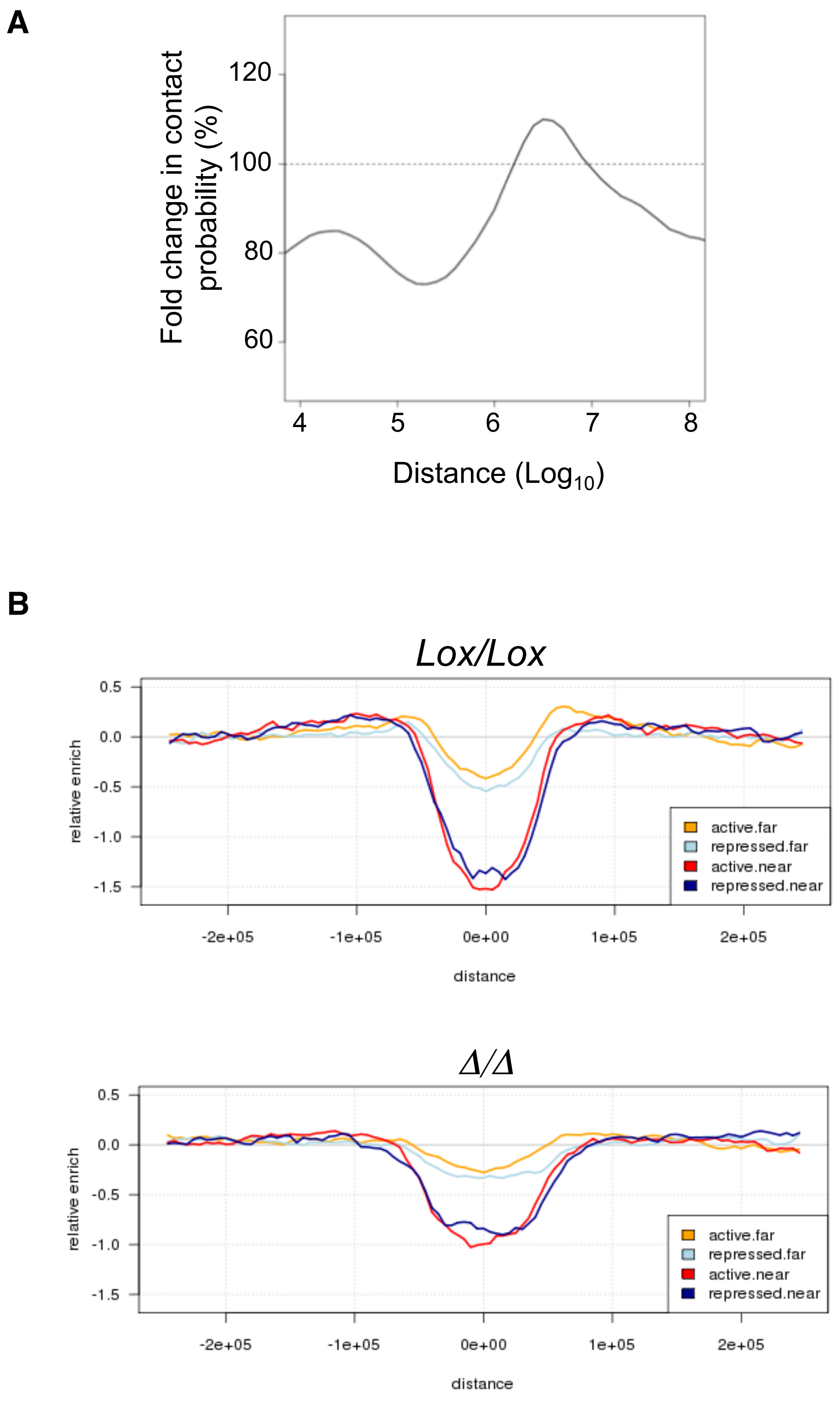

Figure S18: Global contact distribution changes in Rad21-deficient cells. A) Shown is the difference in average contact probability of Rad21-deficient over control ASTs as a function of the logarithm of genomic distance. We note that for ranges closer than 1 Mb ($\log_{10}$ of distance<6), Rad21-deficient cells have significantly fewer contacts on average. This effect is stronger next to cohesin sites, as we show in the main text. B) Average contact intensity of the 80 Kb band of Cohesin/CTCF sites stratified according to domain type (active/repressed) and distance from domain border (threshold of 20 Kb). Note that Cohesin/CTCF sites that are internal to domains also display cohesin-dependent insulatory properties, indicating the existence of an internal sub-division within the identified coarse domains.

Figure S18

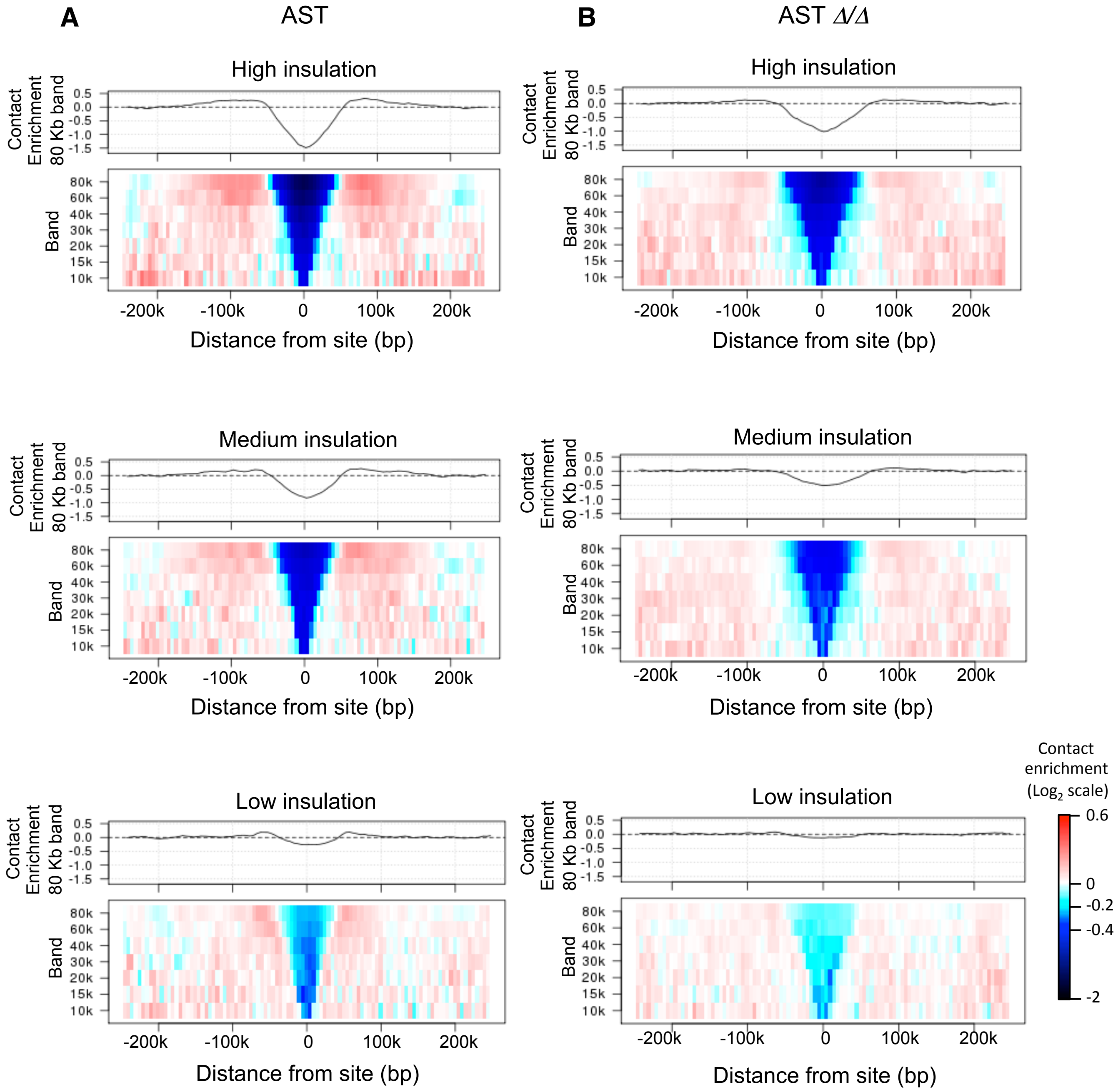

Figure S19: Loss of Rad21 affects a range of cohesin sites. Shown are the insulation diagrams for control ASTs (same as in Figure 1f) as well as diagrams computed for Rad21-deficient ASTs (*Δ/Δ*).

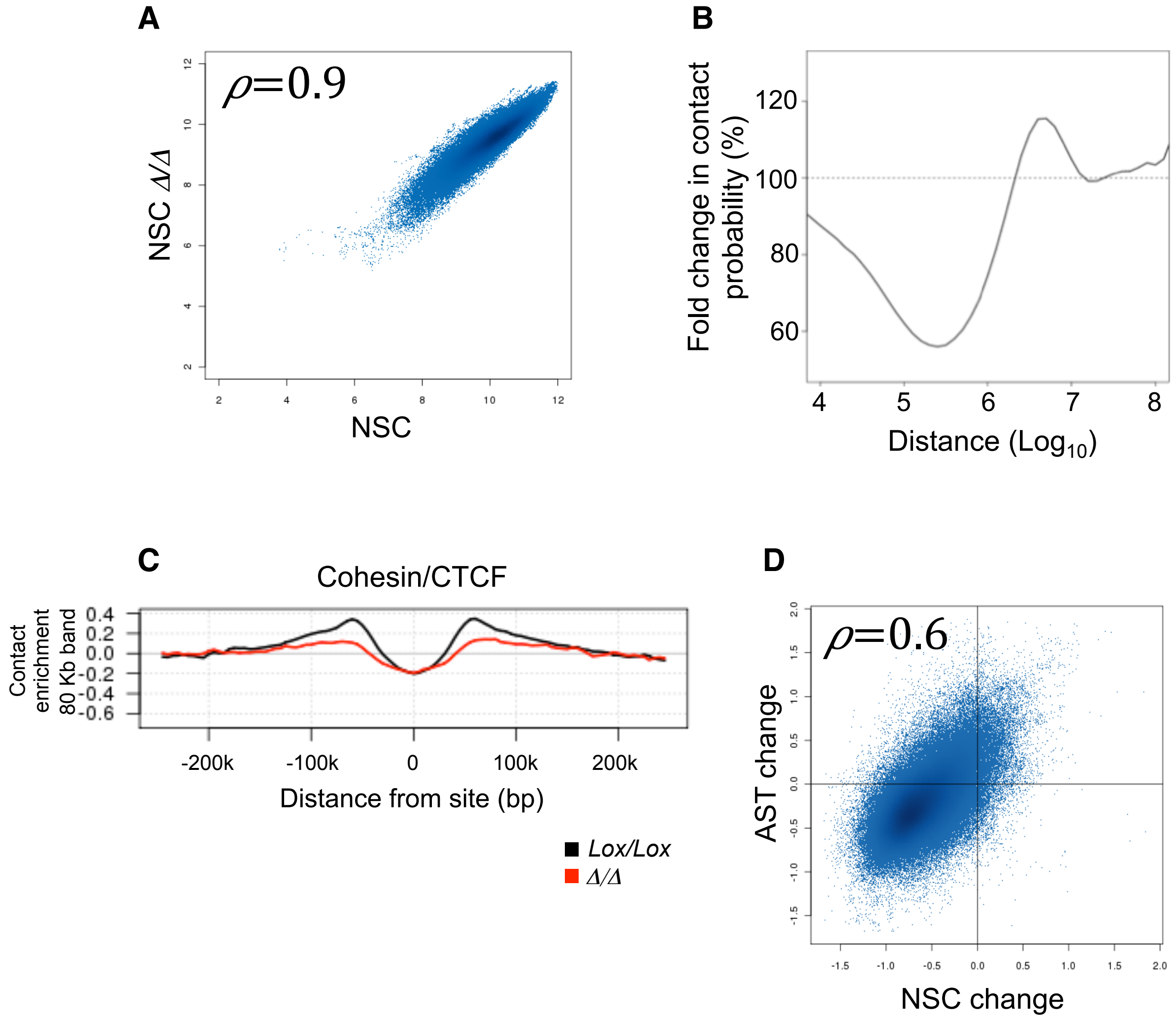

Figure S20: Effects of Rad21-deletion in NSCs are similar to ASTs. A) A comparison of the 160-480 Kb bands between *Rad21*$^{\Delta/\Delta}$ and *Rad21*$^{Lox/Lox}$ Hi-C libraries from NSCs (as in Fig. S17). B) Change in contact probability as a function of distance (as in Fig. S18). C) Change in insulation at cohesin sites (as in Figure 2e). D) A comparison of the effects of Rad21 deletion in ASTs and in NSCs for the 160-480 Kb band, showing that the changes in Hi-C maps which have been prepared from *Rad21*$^{\Delta/\Delta}$ AST and *Rad21*$^{\Delta/\Delta}$ NSCs are correlated.

Figure S20

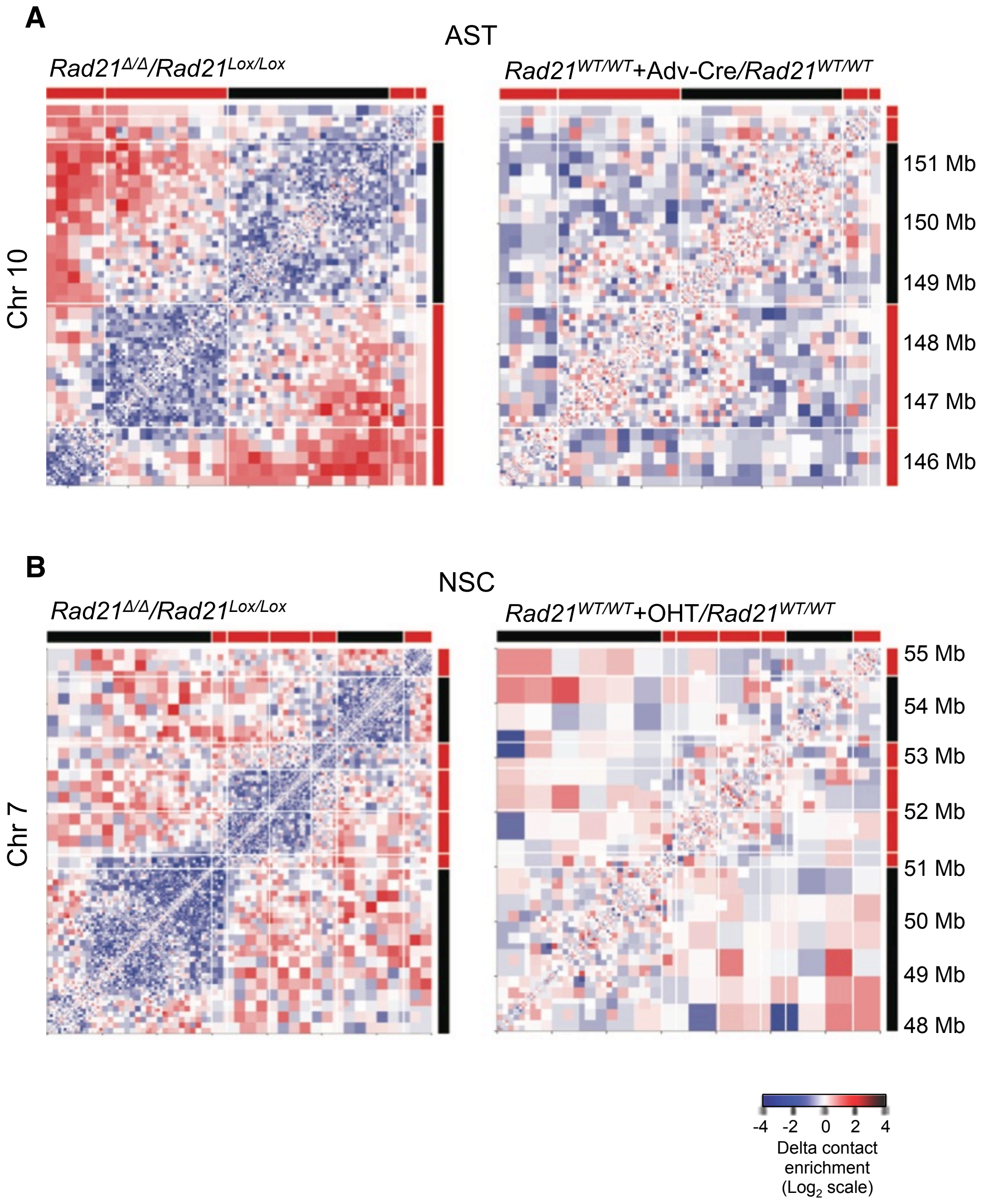

Figure S21: Intra-domain contacts are depleted specifically after Rad21 loss. Hi-C delta maps of regions on chromosomes 10 and 7 in ASTs (A) and NSCs (B), respectively. Note that the characteristic loss of contacts within domains in both NSCs and ASTs upon loss of Rad21 (left) and the minimal effect on domain structure of either OHT or Adenovirus alone in *Rad21*$^{WT/WT}$ cells.

Figure S21

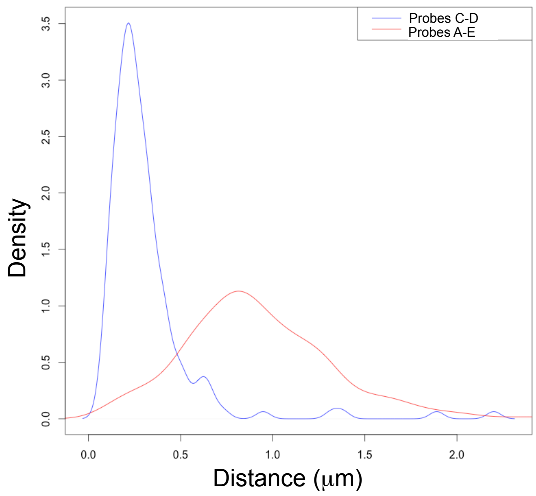

Figure S22: Distribution of distances between FISH signals.
Shown is the distribution of the distances measured for intra-domain FISH probes C-D (blue line) or inter-domain FISH probes A-E (red line) in NSC. Similar observations were made in ASTs.

Figure S22

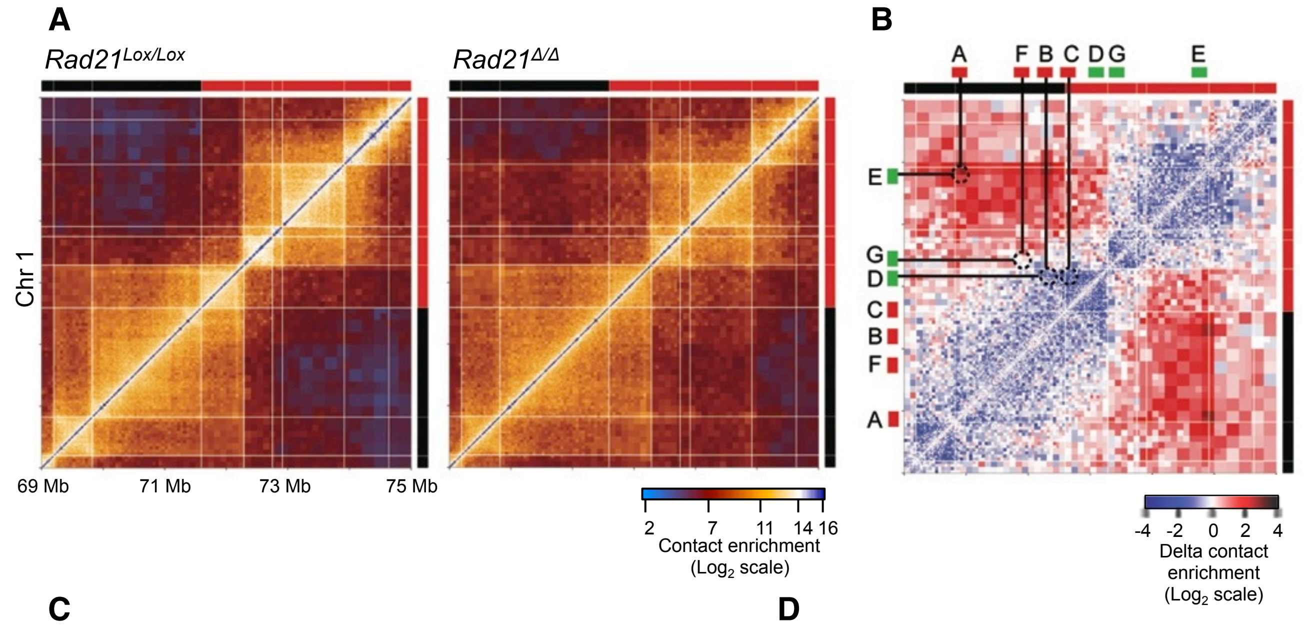

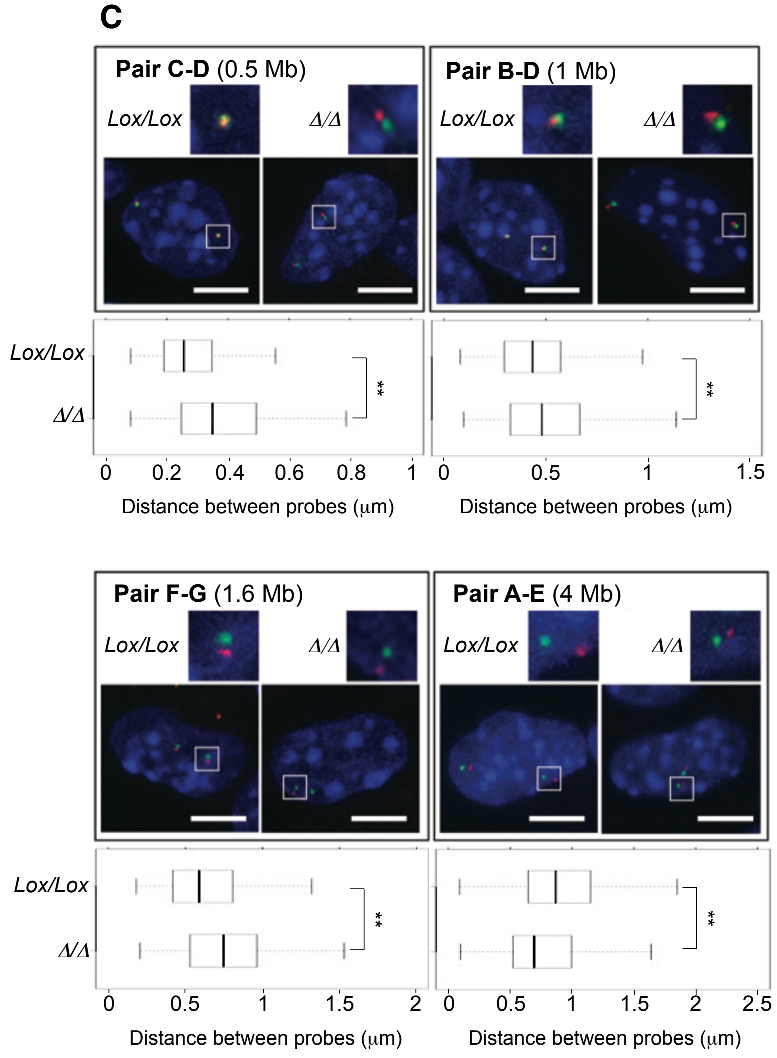

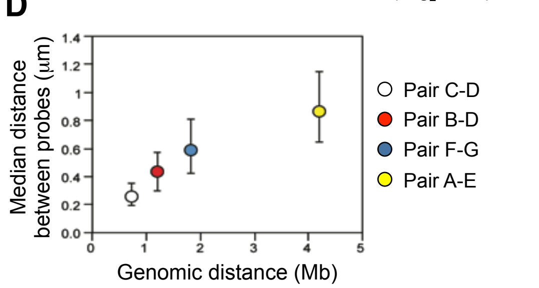

Figure S23: 3D FISH in NSCs. A) Hi-C maps of the region on chromosome 1 analyzed by 3D FISH in *Rad21*$^{Lox/Lox}$ and *Rad21*$^{\Delta/\Delta}$ NSCs. B) Locations of BAC probes along chromosome 1 drawn on a Hi-C delta map. Probe colours correspond to colours in (C). C) Maximum projections from confocal z-stacks of nuclei and Volocity quantifications of three-dimensional inter-probe distances in control (*Lox/Lox*) and cohesin-deficient NSCs (*Δ/Δ*). DNA has been counterstained with DAPI (blue), probes have been labeled with DIG (green) or biotin (red). Genomic distances between probes are indicated in parentheses. White dotted boxes show the regions that have been zoomed in. Whiskers and boxes indicate all and 50% of values respectively. Central bold bars represent the median. Statistical significance has been assessed using a Wilcoxon Rank Sum and Signed Rank Test (Medians: Pair C-D 0.26/0.35 p=0.0001; pair B-D 0.43/0.48 p=0.006; pair F-G 0.59/0.74 p=0.003; pair A-E 0.87/0.69 p=3.6x10^{-6}). Each experiment has been repeated a minimum of two times (n>100/condition, except for probes F-G *Δ/Δ* where n=90). Scale bar = 5 μm. D) The relationship between three-dimensional inter-probe distances and genomic distances.

Figure S23

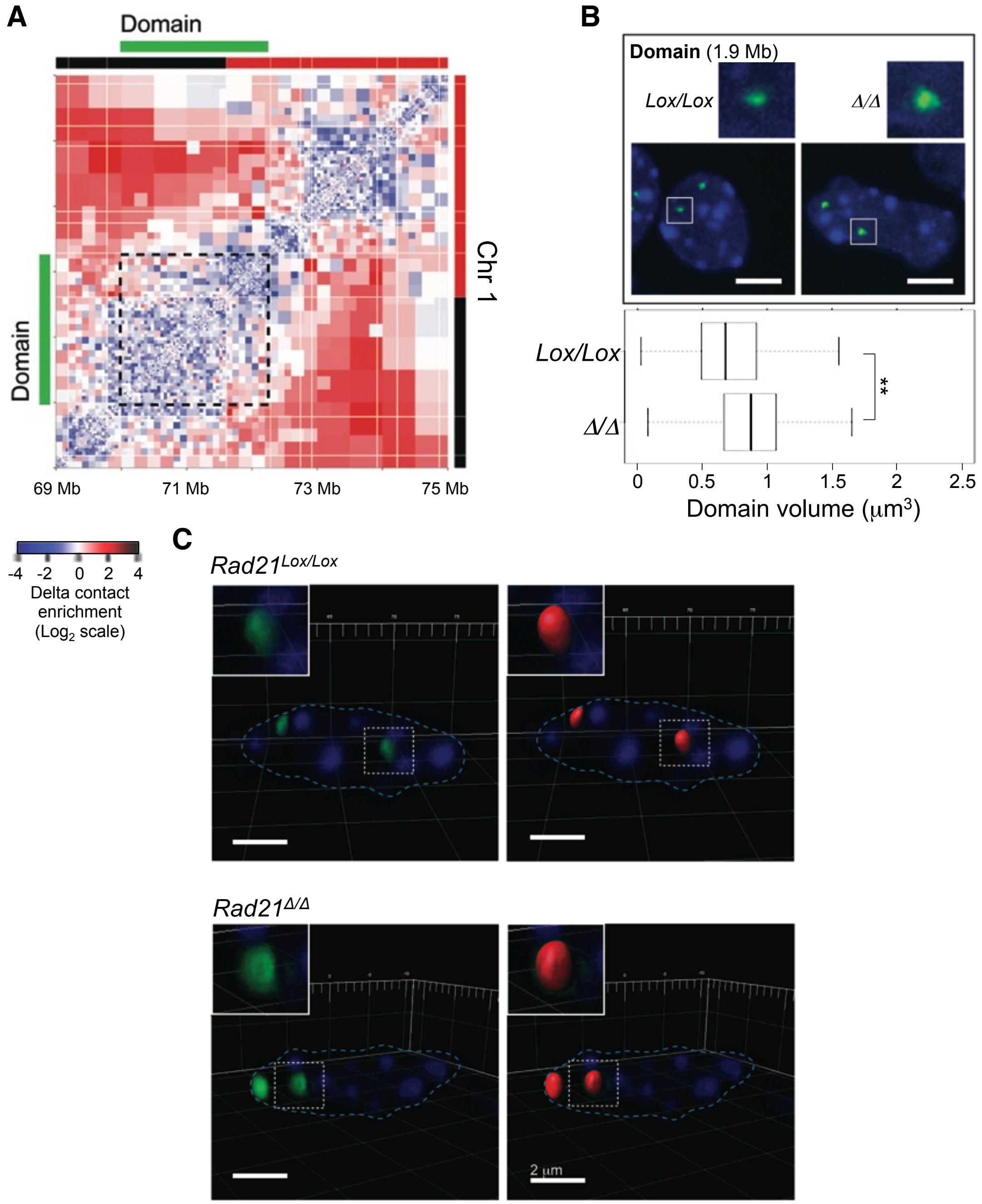

Figure S24: Rad21 loss in ASTs leads to an increase in domain volume. A) The location of six BAC probes (B, C, D, F, H, I) along chromosome 1 used to paint a chromosomal domain, comprised of two neighbouring smaller domains (dashed line) in ASTs. B) Maximum projections from confocal z-stacks and quantification of three-dimensional domain volumes in control (*Lox/Lox*) and cohesin-deficient ASTs (*Δ/Δ*). DNA has been counterstained with DAPI (blue), probes have been labeled with DIG (green). Quantifications were performed using Imaris software as shown in (C). Whiskers and boxes indicate all and 50% of values respectively. Central bars represent the median. Statistical significance has been assessed using a Wilcoxon Rank Sum and Signed Rank Test (Medians: 0.68/0.87, $p=9.2\times10^{-13}$). The experiment has been repeated twice (n>390/condition). Scale bar = 5 μm. C) Three-dimensional representation of the BAC hybridization signal in Imaris in $Rad21^{Lox/Lox}$ and $Rad21^{\Delta/\Delta}$ ASTs (left panels). The rendered volume measured by Imaris has been coloured in red (right panels). Blue dotted lines represent DAPI boundaries. White dotted boxes show the regions that have been zoomed in. Scale bar = 2 μm.

Figure S24

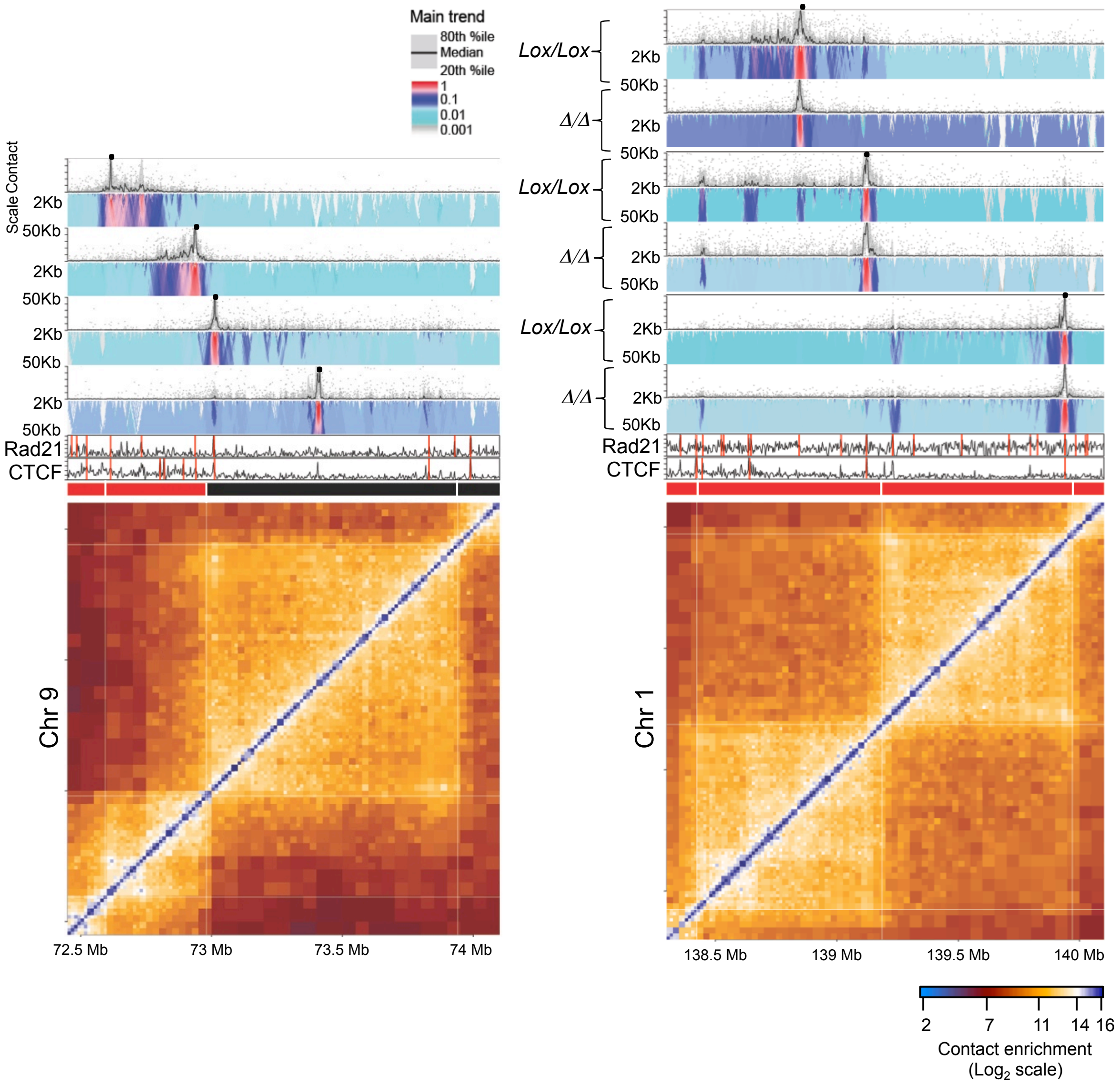

Figure S25: Examples of cohesin/CTCF-based interactions. 4C-seq profiles of additional baits (black spots) anchored at cohesin/CTCF sites, demonstrating preferential and asymmetric cohesin-cohesin interactions. Results from Rad21-deficient cells are also shown for the baits on chromosome 1 and reveal that the specific long-range interactions between cohesin/CTCF sites are lost.

Figure S25

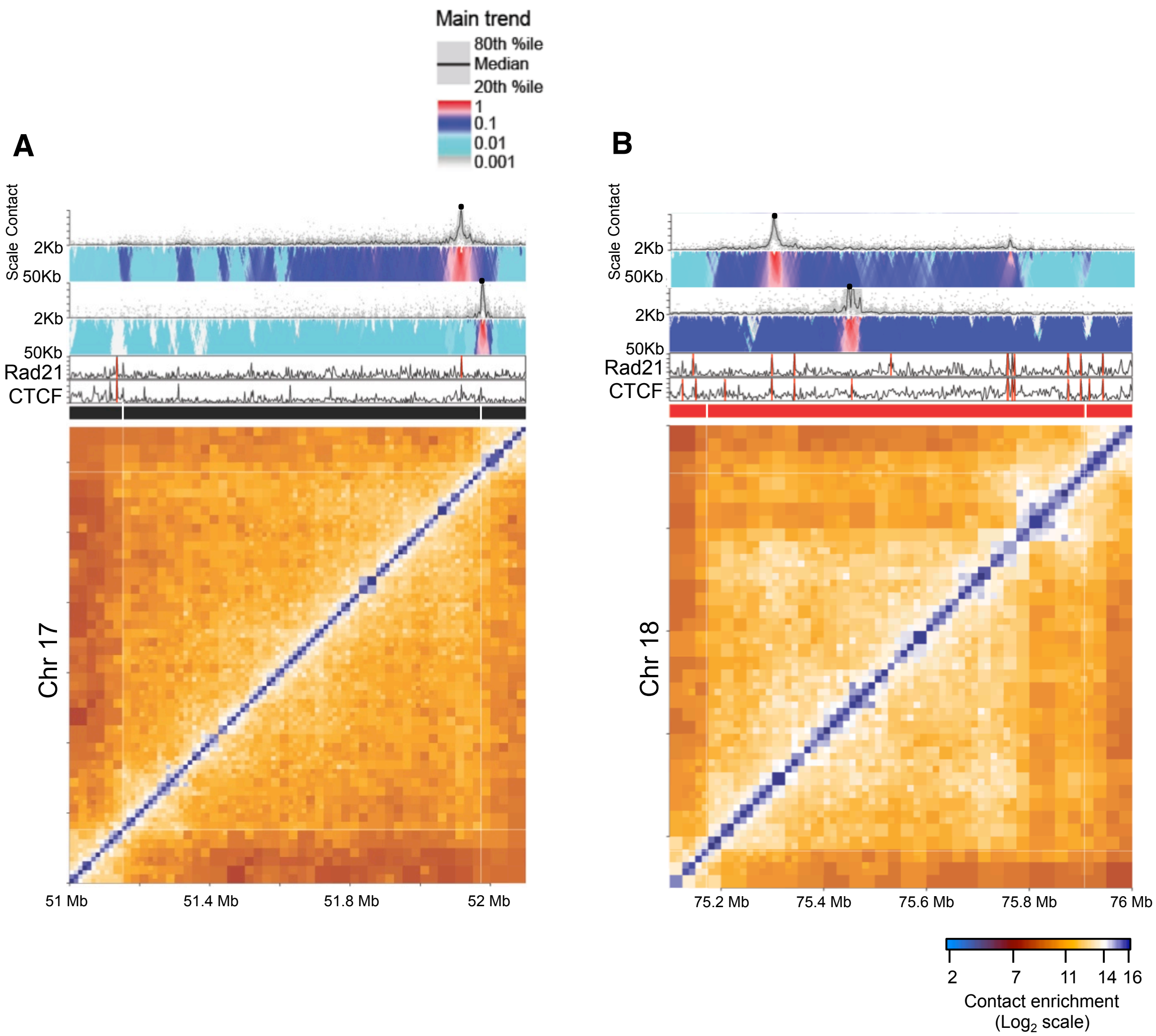

Figure S26: Long-range interactions are only detectable from cohesin-CTCF co-occupied binding sites. A) A 4C-seq map of interactions from a viewpoint (black spot) at a cohesin/CTCF site on chr 17 (top bait) which engages in long-range interactions. In contrast, the viewpoint at a nearby domain boundary, which contains neither a cohesin binding site, nor a strong CTCF site has no specific long-range interactions (bottom bait). B) A 4C-seq profile for a bait anchored at a CTCF-only site (bottom bait). The interaction profile for a nearby cohesin/CTCF bait is shown for comparison (top bait). Note the lack of long-range interactions from the CTCF-only site. The visual representation of contact intensities has been prepared as described in Fig. 3. Also shown are Rad21 and CTCF ChIP tracks and the corresponding Hi-C submatrix.

Figure S26

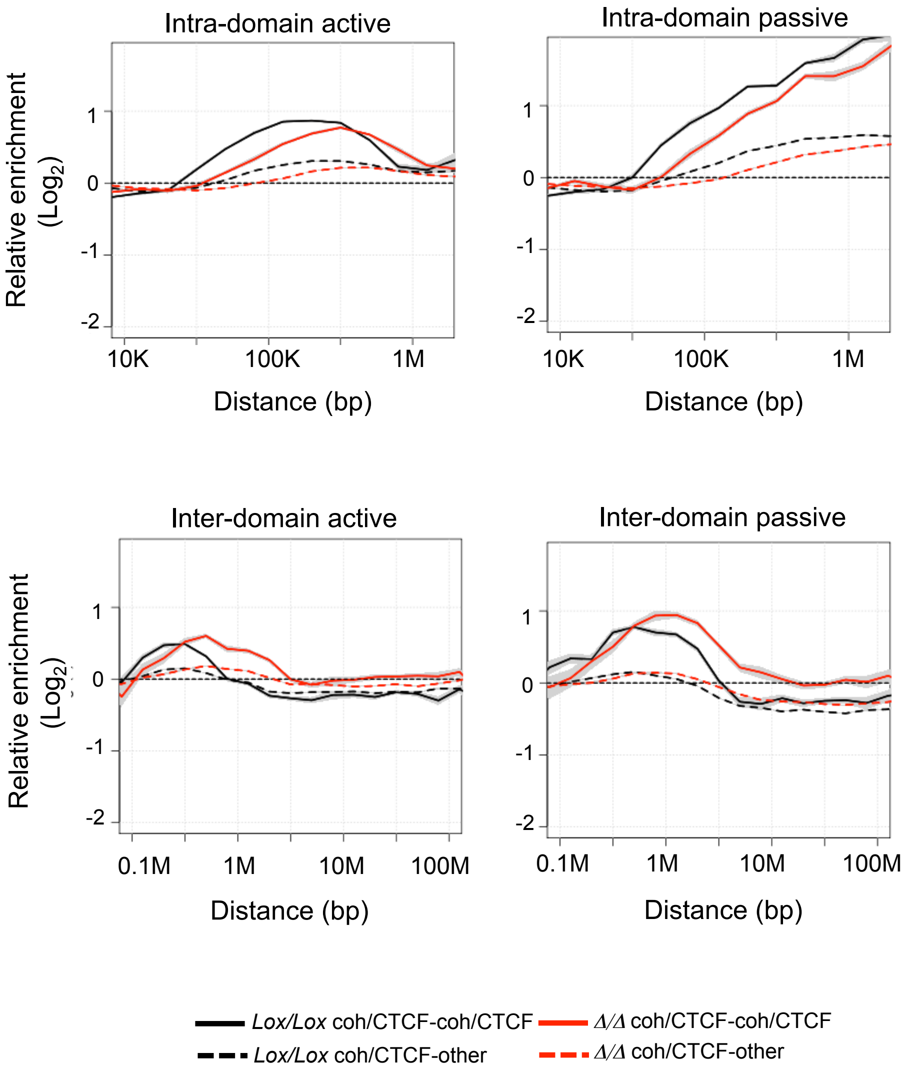

Figure S27: Cohesin-cohesin interactions in NSCs. Shown is the same analysis as in Fig. 3d-e repeated for NSCs.

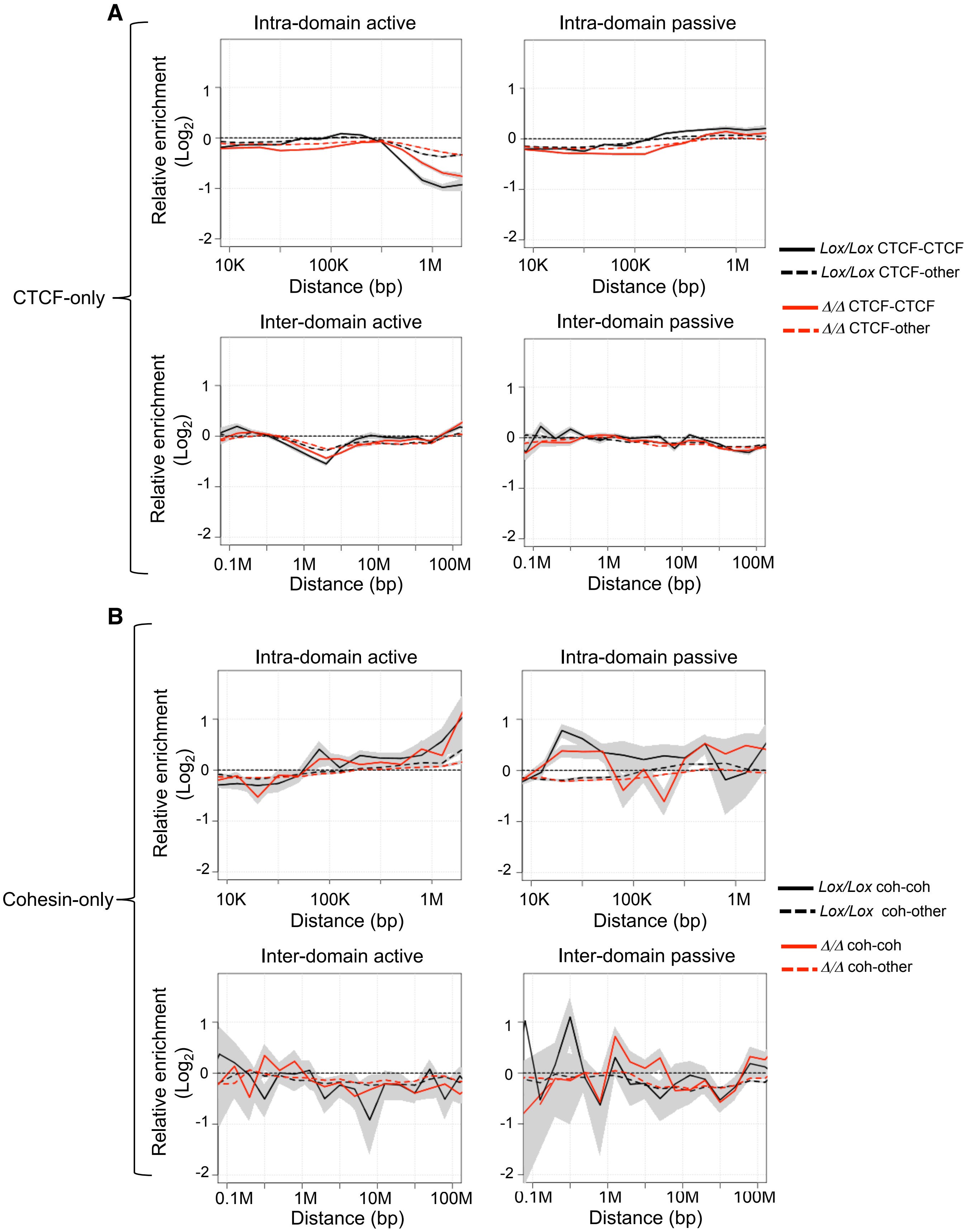

Figure S28: Both CTCF-only and cohesin-only sites do not display distal interactions. Shown is the same analysis as in Fig. 3d-e repeated for (A) CTCF-only sites without strong cohesin (Rad21) binding and (B) Rad21-only sites without strong CTCF binding.

Figure S28

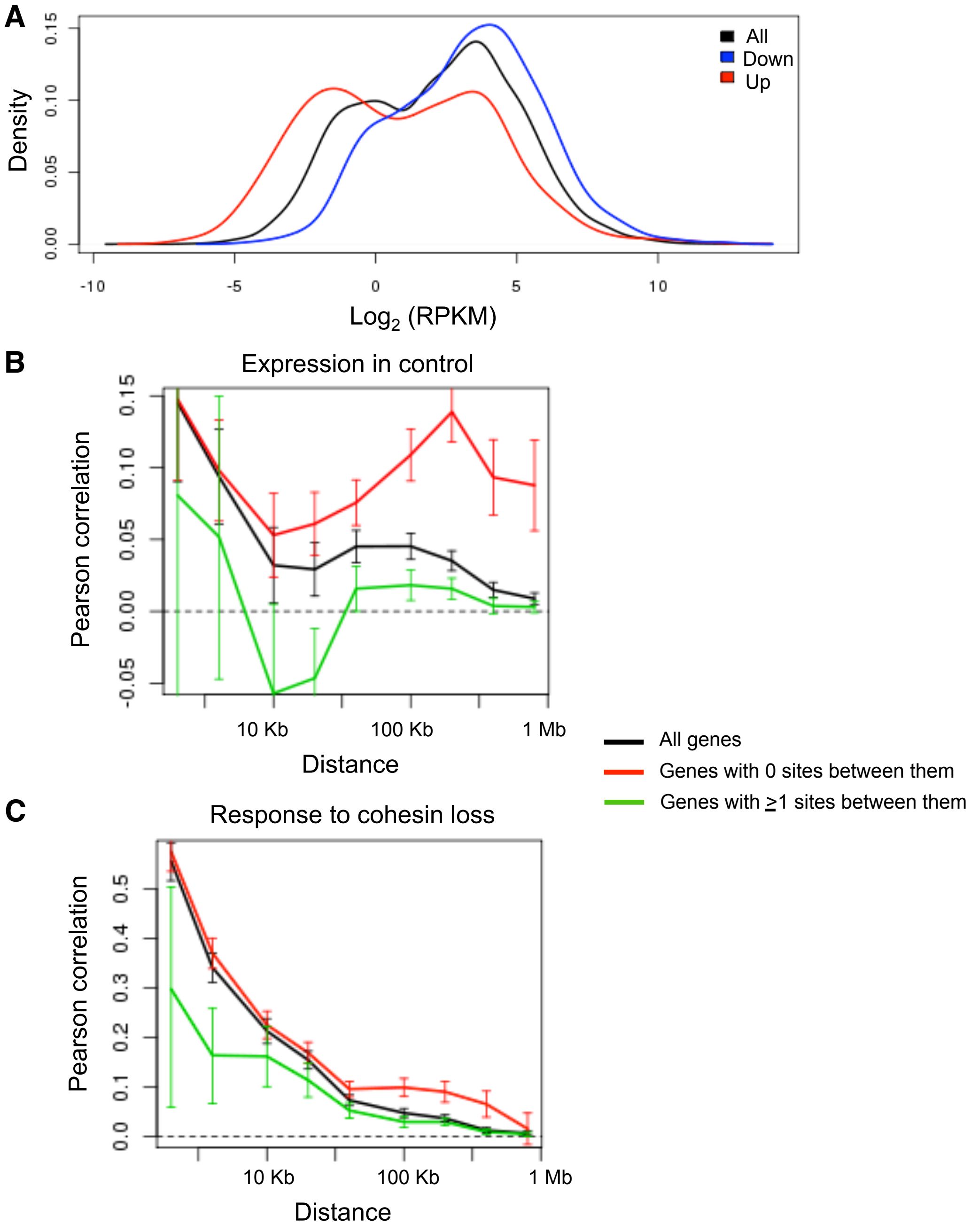

Fig S29: Changes in the transcriptional program of cohesin-deficient cells. A) Cohesin deficiency is correlated with a decay in bimodal expression distribution. Shown is the distribution of wild type transcription levels of genes classified as up- and down- regulated in cohesin-deficient cells. Upon cohesin loss, active genes are repressed and silent genes are activated, supporting cohesin's indirect role in maintaining both repression and activation of genes. B) Mild spatial correlation of wild type transcription levels. Shown is the Pearson correlation of the wild type transcription level (RPKM) of pairs of genes, stratified according to the number of separating cohesin/CTCF sites, for different distance bins (x-axis). A confidence interval was computed based on Fisher's Z transform. The basal spatial correlation is observed at relatively low levels, but is significantly associated with lack of insulatory cohesin/CTCF binding sites. C**)** Genes that are not separated by at least one cohesin/CTCF site respond similarly to Rad21 loss. We define transcriptional response to Rad21 loss as $\log_2$ (KO/WT), where KO and WT are the respective RPKM values for knockout and control cells. Shown is the correlation between the response of adjacent genes, stratified as in (B). Neighbouring genes, which are not separated by at least one cohesin/CTCF site, are significantly correlated in their response. The effect is particularly strong for separating distances lower than 50 Kb, but a significant correlation is observed up to 0.5 Mb.

Figure S29

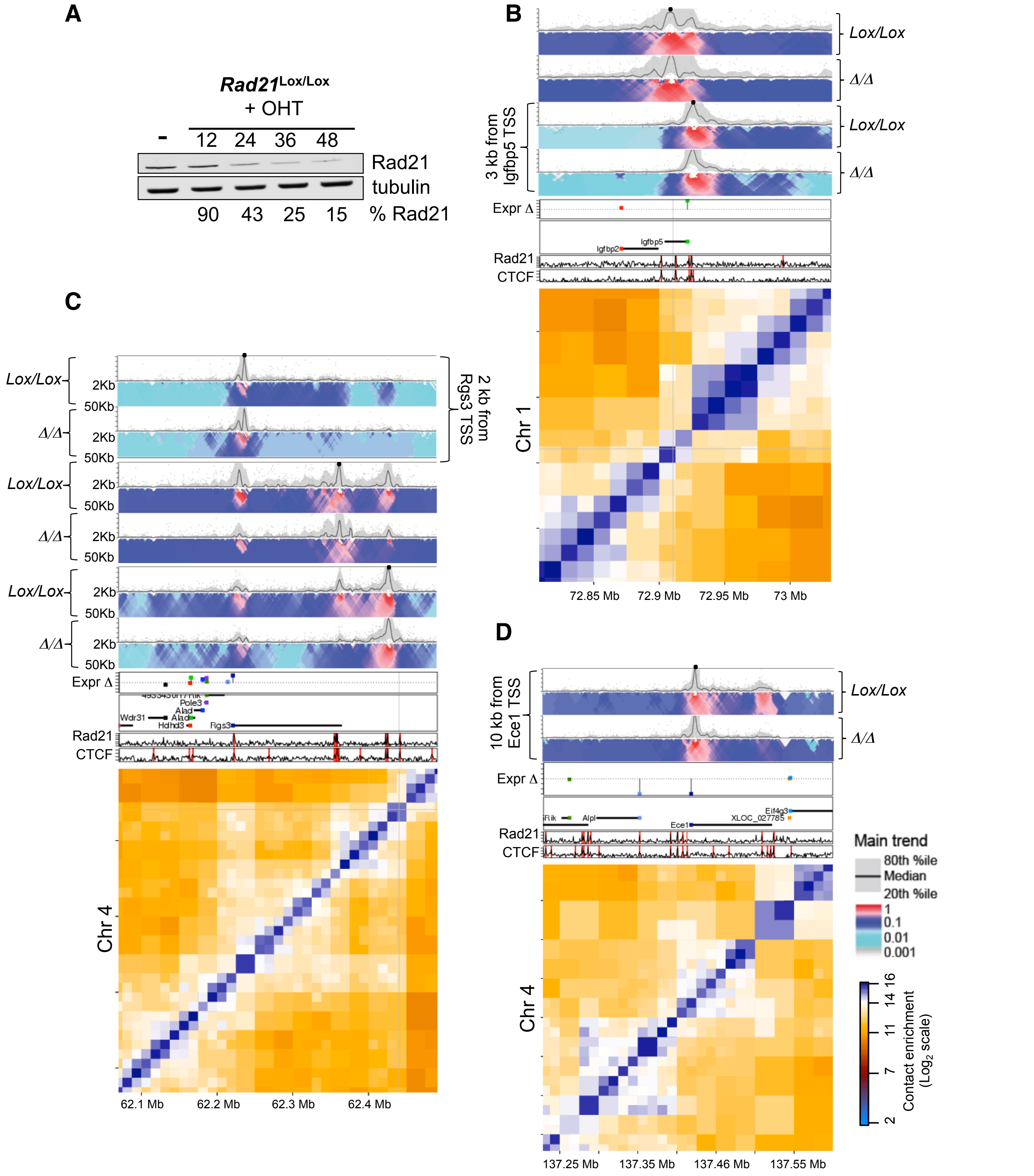

Fig S30: Additional examples of cohesin/CTCF-based structures at deregulated genes. A) Immunoblot analysis of Rad21 protein levels following OHT addition to *Rad21*$^{Lox/Lox}$ cells used for the 4C-seq analysis presented in Fig. 4e. Quantitative Western blot detection was done using the Odyssey imaging system. B) A zoom in of the region surrounding the Igfbp5 gene presented in Fig 4g, showing a specific across-domain long-range interaction that appears near the TSS of Igfbp2 following cohesin loss (bottom bait). C) and D) 4C-seq of baits in the vicinity of Rgs3 and Ece1 in control and knockout cells. Like the Deptor region shown in Fig 4e, we observe that the neighboring cohesin/CTCF-anchored loops around Rgs3 merge after Rad21 loss.

Figure S30

DOWNREGULATED

# Ontology	Hyper Rank	Term Name	Hyper Raw P-Value	Hyper FDR Q-Val	Hyper Fold Enrichment
GO Cellular Component	1	extracellular region	1.60E-08	1.72127E-05	2.426301
GO Cellular Component	2	extracellular region part	2.2414E-06	0.001203631	2.564731
GO Cellular Component	3	extracellular matrix	1.42243E-05	0.005092289	3.178916
Mouse Phenotype	1	abnormal circulating renin level	7.82823E-06	0.057224347	14.91343
Mouse Phenotype	2	fused metatarsal bones	9.23786E-06	0.033764371	21.47535
Mouse Phenotype	3	abnormal protein level	1.63906E-05	0.03993838	2.618945
Mouse Phenotype	4	increased autoantibody level	2.57561E-05	0.047069236	4.971145
Disease Ontology	1	vascular disease	2.31E-07	0.000513004	2.322535
Disease Ontology	2	pre-eclampsia	2.12563E-06	0.002359444	4.238555
Disease Ontology	3	hypertension	5.41023E-06	0.004003571	2.871703
MSigDB Pathway	1	Genes involved in Class A/1 (Rhodopsin-like receptors)	7.04304E-06	0.006197872	5.711528
MSigDB Perturbation	1	Set 'H3K27 bound': genes posessing the trimethylated H3K27 (H3K27me3) mark in their promoters in human embryonic stem cells, as identified by ChIP on chip.	8.79E-09	2.08616E-05	2.854123
MSigDB Perturbation	2	Set 'Suz12 targets': genes identified by ChIP on chip as targets of the Polycomb protein SUZ12 [Gene ID=23512] in human embryonic stem cells.	1.31296E-06	0.00155848	2.631782
MSigDB Perturbation	3	Genes down-regulated in cultured stromal stem cells from adipose tissue, compared to the freshly isolated cells.	1.34233E-06	0.001062234	11.05349

UPREGULATED

# Ontology	Hyper Rank	Term Name	Hyper Raw P-Value	Hyper FDR Q-Val	Hyper Fold Enrichment
GO Biological Process	1	muscle system process	1.28437E-06	0.011023765	8.116221
GO Biological Process	2	positive regulation of multicellular organismal process	1.55704E-06	0.006682046	4.1589
GO Biological Process	3	regulation of muscle contraction	3.69567E-06	0.010573323	10.31061
GO Cellular Component	1	extracellular region	2.18E-09	2.33672E-06	3.059191
GO Cellular Component	2	extracellular region part	2.70E-09	1.45217E-06	3.799632
GO Cellular Component	3	extracellular space	2.78E-07	9.95567E-05	4.017119
Disease Ontology	1	hypertension	9.19E-08	0.00020401	4.110541
Disease Ontology	2	vascular disease	9.97E-07	0.001106136	2.667303
Disease Ontology	3	brucellosis	2.37409E-06	0.00175683	20.0856
MSigDB Perturbation	1	Genes down-regulated in mouse stroma of pancreatic adenocarcinoma zenografts after treatment with HhAntag, a hedgehog (Hh) pathway inhibitor.	1.85E-07	0.000438241	6.111149
MSigDB Perturbation	2	Down-regulated 'cooperation response genes': responded synergystically to the combination of mutant TP53 [Gene ID=7157] and HRAS [Gene ID=3265] in YAMC cells (colon).	4.57154E-06	0.00542642	8.221081
MSigDB Perturbation	3	Genes down-regulated in the luminal B subtype of breast cancer.	3.33683E-05	0.026405416	3.434325
MSigDB Predicted Promoter Motifs	1	Motif TATAAA matches TAF<br>TATA	2.76422E-06	0.001699995	2.768424
InterPro	1	CD59 antigen	3.72306E-06	0.02975098	29.45887
InterPro	2	Ly-6 antigen / uPA receptor -like	8.53208E-06	0.034089926	25.25046
InterPro	3	CD59 antigen, conserved site	1.14797E-05	0.030578201	44.18831

Table S1: Gene Ontology terms enriched in the transcriptional response to cohesin loss.
